# Genetic Analysis Reveals a Hierarchy of Interactions between Polycystin-Encoding Genes and Genes Controlling Cilia Function during Left-Right Determination

**DOI:** 10.1371/journal.pgen.1006070

**Published:** 2016-06-06

**Authors:** Daniel T. Grimes, Jennifer L. Keynton, Maria T. Buenavista, Xingjian Jin, Saloni H. Patel, Shinohara Kyosuke, Jennifer Vibert, Debbie J. Williams, Hiroshi Hamada, Rohanah Hussain, Surya M. Nauli, Dominic P. Norris

**Affiliations:** 1 MRC Harwell, Harwell Science and Innovation Campus, Oxfordshire, United Kingdom; 2 School of Biological Sciences, University of Reading, Whiteknights, Reading, United Kingdom; 3 Diamond Light Source, Beamline B23, Chilton, Didcot, United Kingdom; 4 Chapman University and the University of California, Irvine, Irvine, California, United States of America; 5 Developmental Genetics Group, Graduate School of Frontier Biosciences, Osaka University and CREST, Japan Science and Technology Corporation (JST), Suita, Japan; Stanford University School of Medicine, UNITED STATES

## Abstract

During mammalian development, left-right (L-R) asymmetry is established by a cilia-driven leftward fluid flow within a midline embryonic cavity called the node. This ‘nodal flow’ is detected by peripherally-located crown cells that each assemble a primary cilium which contain the putative Ca^2+^ channel PKD2. The interaction of flow and crown cell cilia promotes left side-specific expression of *Nodal* in the lateral plate mesoderm (LPM). Whilst the PKD2-interacting protein PKD1L1 has also been implicated in L-R patterning, the underlying mechanism by which flow is detected and the genetic relationship between Polycystin function and asymmetric gene expression remains unknown. Here, we characterize a *Pkd1l1* mutant line in which *Nodal* is activated bilaterally, suggesting that PKD1L1 is not required for LPM Nodal pathway activation per se, but rather to restrict *Nodal* to the left side downstream of nodal flow. Epistasis analysis shows that *Pkd1l1* acts as an upstream genetic repressor of *Pkd2*. This study therefore provides a genetic pathway for the early stages of L-R determination. Moreover, using a system in which cultured cells are supplied artificial flow, we demonstrate that PKD1L1 is sufficient to mediate a Ca^2+^ signaling response after flow stimulation. Finally, we show that an extracellular PKD domain within PKD1L1 is crucial for PKD1L1 function; as such, destabilizing the domain causes L-R defects in the mouse. Our demonstration that PKD1L1 protein can mediate a response to flow coheres with a mechanosensation model of flow sensation in which the force of fluid flow drives asymmetric gene expression in the embryo.

## Author Summary

Vertebrates exhibit left-right (L-R) asymmetry in positioning and patterning their internal organs and associated vasculature; abnormal L-R asymmetry can result in birth defects such as congenital heart disease. The earliest known event in mammalian L-R patterning is a leftward fluid flow across a transient embryonic structure termed the node. This ‘nodal flow’ is driven by the action of motile cilia, hair-like organelles protruding from the cell surface within the node. Nodal flow is sensed by crown cells that surround the node; this requires immotile primary cilia and the putative cation channel Polycystin-2 (PKD2). A second Polycystin protein, PKD1L1, is implicated in this pathway. We describe two principle findings: a genetic hierarchy in cilia-motility genes act upstream of Polycystin-encoding genes and in which *Pkd1l1* acts upstream of, and likely represses *Pkd2*. We further demonstrate that PKD1L1 is sufficient to mediate a flow-induced Ca^2+^ response in cultured cells, and that an extracellular PKD domain is critical for both flow detection and proper L-R patterning. Together, these findings reveal a genetic pathway operating at the level of flow sensation and demonstrate that PKD1L1 is able to act to elicit flow-induced Ca^2+^ signals, thereby supporting the mechanosensation model of nodal flow sensation.

## Introduction

The internal organs and vasculature of vertebrates are highly asymmetrical between left and right. For example, the heart and stomach become positioned towards the left side while the lungs undergo asymmetric branching, giving rise to different numbers of lobes on the left and right sides. Severe situs defects, such as the loss of concordance in positioning between different organs, called heterotaxia [1], are not usually compatible with survival. Moreover, situs defects frequently manifest as congenital heart malformations [2, 3] and therefore represent a major healthcare concern.

During mouse embryogenesis, left-right (L-R) symmetry is broken by leftward fluid flow across the surface of the node, a transient embryonic cavity filled with extracellular fluid [4, 5]. Leftward (or nodal) flow is generated by the clockwise motion (when viewed ventrally) of 200–300 motile monocilia, referred to here as nodal cilia. The core logic of this nodal flow model, that symmetry is broken by cilia-driven leftward flow, is now known to apply to other species including the teleost fish, zebrafish and medaka fish, as well as the frog *Xenopus laevis* [6].

As a result of nodal flow, asymmetries in gene expression emerge around the node in peripherally-located crown cells at the lateral edges of the pit. *Cerl2* (Mouse Genome Informatics–*Dand5*) becomes expressed in crown cells with a right-sided bias (R>L) by the late headfold (LHF) to early somite stages [7, 8]. CERL2 then asymmetrically represses Nodal signaling, resulting in an R<L asymmetry in crown cell NODAL activity [9]. This bias is transmitted to the lateral plate mesoderm (LPM) where a cascade consisting of NODAL, its’ feedback repressor LEFTY2, and the transcription factor PITX2 (henceforth, the ‘Nodal cascade’) is established on the left side only [10, 11]. This left-restricted Nodal cascade is critical for proper organ L-R asymmetry and is highly conserved throughout vertebrates and some invertebrates [12]. Importantly, expression of *Lefty1* at the midline in the prospective floor plate (PFP) inhibits left-sided NODAL signals from spreading and activating the Nodal cascade on the right side of the mouse embryo [13, 14], thereby maintaining unilateral left-sided pathway activity.

How nodal flow in the node drives downstream gene expression asymmetries in crown cells has remained unanswered. Previous studies have suggested roles for Polycystin-2 (PC-2, PKD2 or TRPP2) and Polycystin 1-like 1 (PKD1L1) in the response to nodal flow [15–20]. PKD2 is a six-pass transmembrane protein that acts as a non-selective cation channel [21], while PKD1L1 is an eleven-pass transmembrane protein which is argued to be sensory. PKD1L1 is expressed within the node and interacts with PKD2 [16, 17], leading to the notion that PKD1L1-PKD2 complexes within nodal cilia act as sensors of nodal flow [22]. However, the nature of the asymmetric signal that engages putative PKD1L1-PKD2 sensory complexes is not known. One proposal, the morphogen model, argues that the concentration of a chemical determinant becomes asymmetric in response to flow, thereby initiating a left-sided pathway [5, 23]. A second model, historically called the ‘two-cilia hypothesis’ (here referred to as the mechanosensation model), posits that the force of flow within the node is sensed on the left side, where it is likely to be stronger, thereby initiating events on the left that ultimately activate *Nodal* [18].

Polycystin-1 family members are known to bind to Polycystin-2 family members (TRPPs) and form receptor-channel complexes in contexts beyond L-R patterning. For example, PKD1 and PKD2 form complexes that are thought to sense urine flow and elicit downstream Ca^2+^ signals in the kidney; defects in this process may underlie autosomal dominant polycystic kidney disease (ADPKD) [24–27]. Moreover, the relative dosage of PKD1 and PKD2 influences the activity of stretch-activated ion channels (SACs) to regulate the sensation of pressure and control the arterial myogenic tone [28]. In this context, PKD1 and PKD2 act in an antagonistic fashion to control downstream events. Thus, diverse roles exist in the sensation of forces by PKD1/PKD2 in epithelial and endothelial cells. Polycystin complexes have also been documented to respond to other kinds of stimuli. PKD1L3/PKD2L1, for example, assemble to form an acid-sensing ion channel complex [29, 30] which plays a role in sour taste responses [31]. Thus, pairs of polycystin proteins sense a variety of stimuli via genetic and molecular mechanisms that are not well understood.

Here, we elaborate a genetic cascade acting downstream of nodal flow in L-R patterning that results in initiation of left-sided *Nodal*. We describe a *Pkd1l1* mutant in which the Nodal cascade is bilaterally activated, suggesting that *Pkd1l1* is required not for *Nodal* activation but to restrict *Nodal* to the left side. Moreover, *Pkd1l1* acts genetically upstream of *Pkd2* and downstream of flow; this regulates *Cerl2* asymmetry in a cilia-dependent fashion. Using artificial flow in a cell culture system, we find that PKD1L1 can mediate a response to fluid flow, initiating a Ca^2+^ signaling event upon the onset of flow. Finally, we demonstrate that an extracellular polycystic kidney disease (PKD) domain is critical for PKD1L1 function: a destabilizing mutation in the domain results in a lack of the flow-induced Ca^2+^ response in cultured cells and L-R patterning abnormalities in the mouse. This provides evidence that PKD1L1 mediates sensation of fluid flow in L-R patterning.

## Results

**Fig 1:**
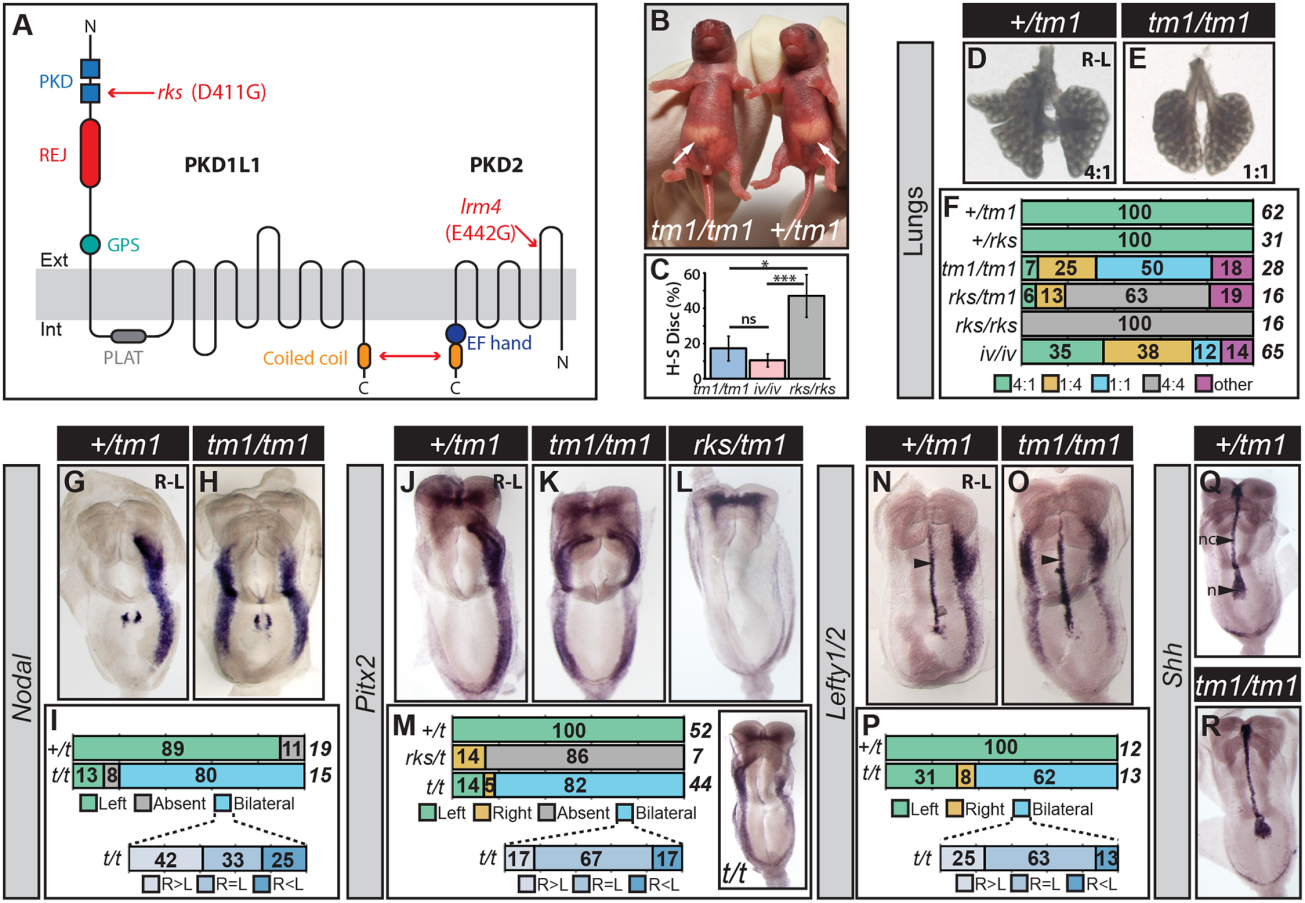
Phenotyping of *Pkd1l1*^*tm1/tm1*^ Mutants. (A) Schematic diagram of PKD1L1 and PKD2 showing protein domains and the nature of the *Pkd1l1*^*rks*^ and *Pkd2*^*lrm4*^ point mutations. The double headed red arrow denotes the site of interaction between PKD1L1 and PKD2. PKD—Polycystic Kidney Disease; REJ—Receptor for Egg Jelly; GPS—G-protein Coupled Receptor Proteolytic Site; PLAT—Polycystin-1, Liopoxygenase, Alpha-Toxin. (B) *Pkd1l1*^*tm1/tm1*^ and sibling control showing reversed and normal situs, respectively. White arrows indicate stomach position. (C) Heart-stomach discordance (H-S Disc.) in *Pkd1l1*^*tm1/tm1*^, *Dnah11*^*iv/iv*^ and *Pkd1l1*^*rks/rks*^ mutants scored at E13.5. Normally, the heart apex and stomach are positioned to the left. H-S Disc. is defined as the heart apex and stomach being on opposite sides. ns—not significant; *—p<0.05; **—p<0.001, Fisher’s Exact Test applied. (D-F) Lung situs assessed at E13.5 for embryos of the indicated genotypes with the ratio of lung lobes between left and right sides given. The percentage and total numbers of embryos showing each phenotype are indicated in *(F)*. (G-P) Expression patterns of *Nodal*, *Pitx2*, and *Lefty1/2* in embryos at E8.5 of the indicated genotypes, with the percentage number of embryos exhibiting each phenotype and the total number given. Embryos exhibiting bilateral marker expression are further categorized by whether they show equal or biased expression between the left and right sides. The inset in *(M)* shows a *Pkd1l1*^*tm1/tm1*^ embryo with bilateral *Pitx2* expression but with a right-sided bias. Arrowheads in *(N)* and *(O)* indicate midline *Lefty1* expression. *t* is shorthand for *Pkd1l1*^*tm1*^. (Q-R) Sonic hedgehog (*Shh*) expression in the node (n) and notochord (nc) at E8.5.

We have previously analyzed a mouse *Pkd1l1* point mutant, called *Pkd1l1*^*rks*^ (Fig 1A) [16]. *Pkd1l1*^*rks/rks*^ homozygotes fail to activate the LPM Nodal cascade and exhibit morphological L-R defects similar to those displayed by both *Pkd2*^*–/–*^ null mutants and *Pkd2*^*lrm4/lrm4*^ point mutants (Fig 1A) [16, 19, 32]. However, since *Pkd1l1*^*rks*^ is a point mutant, we set out to address the impact on L-R development of a distinct *Pkd1l1* allele, namely the targeted mutation *Pkd1l1*^*tm1Lex*^ (here named *Pkd1l1*^*tm1*^), which is likely a loss-of-function allele (S1 Fig) [33].

### *Pkd1l1*^*tm1/tm1*^ and *Pkd1l1*^*rks/rks*^ Mutants Exhibit Discrete Abnormalities

When assessed on the same genetic background, *Pkd1l1*^*tm1/tm1*^ and *Pkd1l1*^*rks/rks*^ mutants exhibited distinct phenotypes. First, while the homozygous *Pkd1l1*^*rks*^ mutation is lethal at embryonic day (E) 14.5–15.5 [16], we found that a proportion of *Pkd1l1*^*tm1/tm1*^ mutants survived until adulthood (S2A and S2B Fig). Surviving mutants either exhibited normal (57%) or reversed (43%) situs based on stomach position in neonates (Fig 1B). Second, though the laterality of heart and stomach was randomized in both *Pkd1l1*^*tm1/tm1*^ and *Pkd1l1*^*rks/rks*^ mutants at E13.5 (S2C–S2E Fig), the latter exhibited a significantly higher level of discordance between the two organs, reflecting a heterotaxic phenotype (Fig 1C). Third, whilst all *Pkd1l1*^*rks/rks*^ mutants exhibited right lung isomerism, where four lung lobes develop on both the left and right sides [16], the majority of *Pkd1l1*^*tm1/tm1*^ embryos displayed the complete opposite phenotype of bilaterally mono-lobed lungs, called left lung isomerism (Fig 1D–1F). This phenotype was not completely penetrant; some *Pkd1l1*^*tm1/tm1*^ mutants exhibited other lung lobation patterns, though never right isomerism (Fig 1F). In conclusion, the *Pkd1l1*^*tm1*^ allele has a strikingly different impact on both survival and L-R patterning compared to the *Pkd1l1*^*rks*^ point mutation.

### *Pkd1l1*^*tm1/tm1*^ Mutants Bilaterally Activate the Nodal Cascade

To pinpoint the molecular causes of the L-R defects in *Pkd1l1*^*tm1/tm1*^ mutants, we examined L-R marker gene expression by mRNA whole mount in situ hybridization (WISH). In control embryos, *Nodal* was nearly always expressed in the left LPM between the 3–7 somite stages (ss) (Fig 1G and 1I), as expected. In contrast, *Nodal* was most often expressed bilaterally in *Pkd1l1*^*tm1/tm1*^ mutants (Fig 1H and 1I). Similarly, *Pitx2* and *Lefty2*, both downstream targets of Nodal signaling in the LPM, were most frequently expressed bilaterally in *Pkd1l1*^*tm1/tm1*^ embryos (Fig 1J, 1K, 1M and 1P). Of those embryos that exhibited bilateral activity of the Nodal cascade, most displayed similar levels and extents of *Nodal/Lefty2/Pitx2* expression on both sides, but some embryos exhibited a left or right bias (Fig 1I, 1M and 1P). Whilst the bilateral induction of the Nodal cascade in *Pkd1l1*^*tm1/tm1*^ embryos explains the high level of left lung isomerism, the fact that some embryos exhibit slight, though randomized, asymmetries in this bilaterality might serve to explain why we find a relatively high incidence of normal, inverted, and other lung lobation patterns (50% in total; Fig 1F) that depart from the left isomerism predicted by truly bilateral Nodal signals. In summary, most *Pkd1l1*^*tm1/tm1*^ mutant embryos exhibit bilateral activation of the Nodal cascade, representing another striking difference to *Pkd1l1*^*rks/rks*^ mutants that display the opposite phenotypes of loss of LPM Nodal activity [16].

As a corollary of these results, we generated *Pkd1l1*^*rks/tm1*^ trans-heterozygotes to further understand the differences between the *Pkd1l1*^*rks*^ and *Pkd1l1*^*tm1*^ alleles. The majority of *Pkd1l1*^*rks/tm1*^ embryos exhibited right lung isomerism and loss of LPM *Pitx2* expression (Fig 1F, 1L and 1M), suggesting that the *Pkd1l1*^*rks*^ allele is dominant over *Pkd1l1*^*tm1*^. We discuss the nature of the *Pkd1l1*^*rks*^ allele further in the **Discussion**.

Bilateral Nodal cascade activity in L-R mutants can result from midline defects [5, 34], including the failure to express *Lefty1* in the floor plate which constitutes a molecular ‘midline barrier’ [13]. The node and notochord were clearly specified in *Pkd1l1*^*tm1/tm1*^ mutants, as evidenced by normal Sonic hedgehog (*Shh*) expression (n = 6/6 embryos) (Fig 1Q and 1R). *Lefty1* was also expressed at the midline of *Pkd1l1*^*tm1/tm1*^ mutants; indeed *Lefty1* expression appeared more intense than in control embryos, perhaps owing to bilaterally high NODAL activity inducing *Lefty1* in both the left and right sides of the floor plate (Fig 1N and 1O). Thus, the *Lefty1* midline barrier is formed in *Pkd1l1*^*tm1/tm1*^ mutants. Since *Pkd1l1*^*tm1*^ appears to be loss-of-function (hypomorphic or null), these data argue that rather than being required for activation of the LPM Nodal cascade, *Pkd1l1* is instead needed to restrict Nodal pathway activity to the left side of the embryo in a mechanism that is independent of the midline barrier.

### *Pkd1l1* Acts Downstream of Nodal Flow

**Fig 2:**
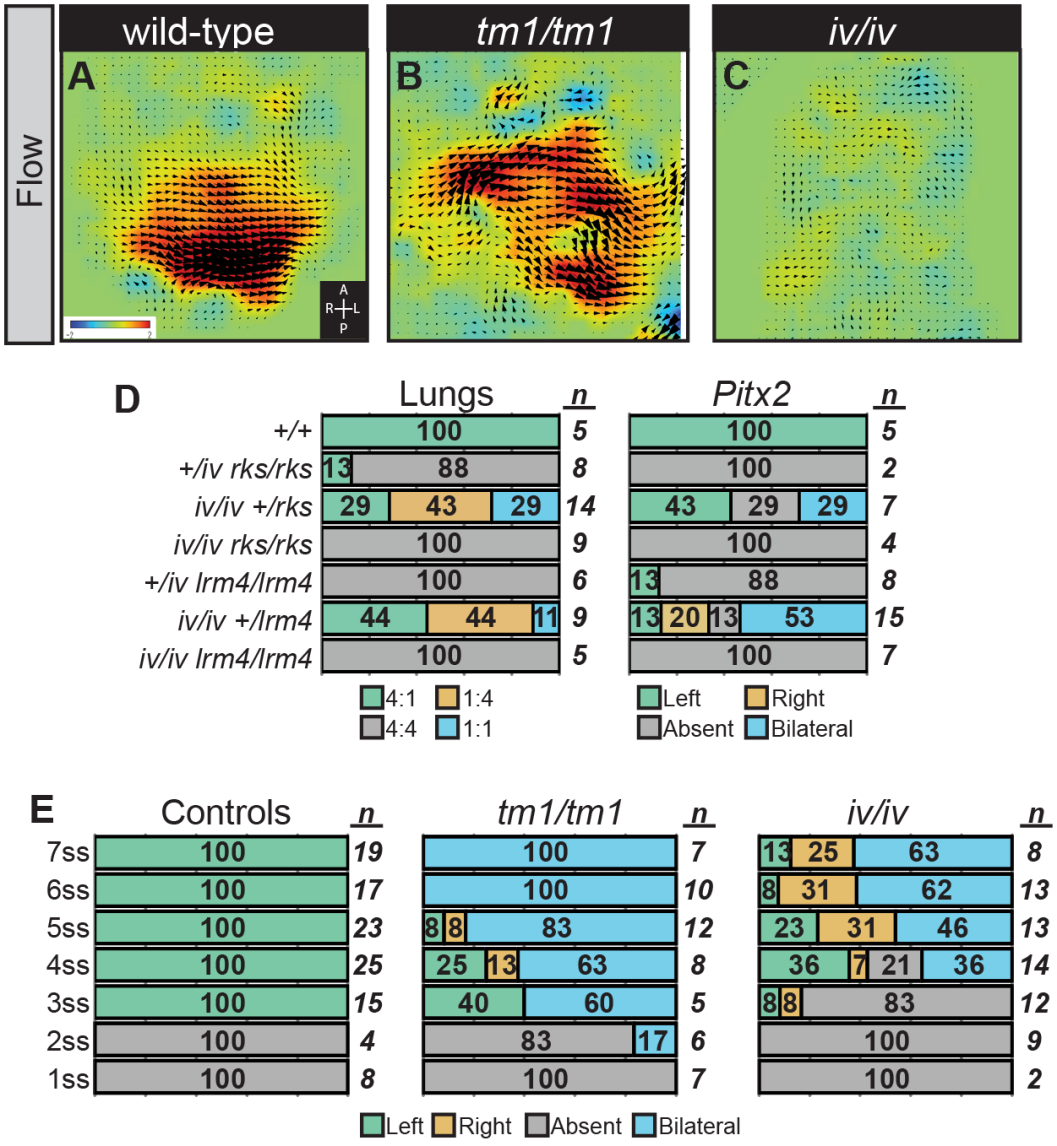
The Relationship Between Nodal Flow and *Pkd1l1/Pkd2* Function. (A-C) Nodal flow in embryos of indicated genotypes was examined at the 1–3 somite stages by means of PIV analysis. Flow was normal in *Pkd1l1*^*tm1/tm1*^ mutants and wild-type controls but was absent in *Dnah11*^*iv/iv*^ mutants. Black arrowheads denote the direction and speed of flow at that position while the false coloring indicates the direction and magnitude of the flow. Red indicates leftward and blue rightward fluid movements. (D) Lung situs (assessed at E13.5) and *Pitx2* expression (assessed at E8.5) for embryos of the indicated genotypes, with the percentage of embryos exhibiting each phenotype and the total number given. (E) *Pitx2* expression for *Pkd1l1*^*tm1/tm1*^, *Dnah11*^*iv/iv*^ and control embryos for each of the 1–7 somite stages. The onset of *Pitx2* expression is delayed in *Dnah11*^*iv/iv*^ mutants but not in *Pkd1l1*^*tm1/tm1*^ embryos.

Bilateral establishment of the Nodal cascade in both the right and left LPM can be caused by aberrant or absent nodal flow [35, 36]. We therefore imaged the node using differential interference contrast (DIC) microscopy and found that nodal cilia rotated normally, at around 12 Hz, in both *Pkd1l1*^*tm1/tm1*^ mutants and wild-type controls (S3 Fig). We next assessed flow directly by immersing embryos in fluorescent beads, imaging the node cavity, then performing particle image velocimetry (PIV) analysis [37] to obtain a map of flow movements. This revealed that directional leftward flow was indeed generated both in control embryos and *Pkd1l1*^*tm1/tm1*^ mutants (Fig 2A, 2B and S4 Fig). Thus, the Nodal cascade is induced bilaterally even in the presence of normal nodal flow in *Pkd1l1*^*tm1/tm1*^ mutants.

Given that nodal flow is present in *Pkd1l1*^*tm1/tm1*^ embryos while LPM *Nodal* expression is predominantly bilaterally activated, we predicted that *Pkd1l1* acts downstream of flow. Indeed, this would be expected for molecules required for flow-sensing. In order to determine the genetic control and the order of these events we assessed epistasis between *Pkd1l1*^*rks*^ and *Dnah11*^*iv*^; *Dnah11*^*iv*^ disrupts an axonemal dynein heavy chain, resulting in immotile nodal cilia and loss of nodal flow (Fig 2C and S4 Fig) [35]. At E13.5 (lung situs assessed) and E8.5 (*Pitx2* expression assessed), *Pkd1l1*^*rks/rks*^;*Dnah11*^*iv/iv*^ double mutants exhibited right lung isomerism and loss of LPM *Pitx2*, respectively, and therefore phenocopied *Pkd1l1*^*rks/rks*^ mutants rather than *Dnah11*^*iv/iv*^ mutants (Fig 2D and S1 Table). This demonstrates that the *Pkd1l1*^*rks/rks*^ phenotype still manifests in the absence of nodal flow. Similar results were obtained when we assessed epistasis between *Dnah11*^*iv*^ and *Pkd2*^*lrm4*^; in this experiment, *Pkd2*^*lrm4/lrm4*^;*Dnah11*^*iv/iv*^ double mutants phenocopied *Pkd2*^*lrm4/lrm4*^ and not *Dnah11*^*iv/iv*^ (Fig 2D and S1 Table). Thus, genetically ablating flow, which is otherwise present, does not alter the L-R phenotype of either *Pkd1l1*^*rks/rks*^ or *Pkd2*^*lrm4/lrm4*^ embryos. This demonstrates that the roles of *Pkd1l1* and *Pkd2* are downstream of nodal flow, supporting the notion that these genes mediate the sensation of flow. We did not generate *Pkd1l1*^*tm1/tm1*^;*Dnah11*^*iv/iv*^ double mutants because the phenotypes of the single mutants are highly similar (S2 Fig), so an analysis of double mutant embryos would be uninformative.

### Induction of the Nodal Cascade Is Delayed in Embryos Lacking Flow but Not in *Pkd1l1*^*tm1/tm1*^ Mutants

If the response to flow is mediated through PKD1L1, it might be expected that loss of nodal flow (*Dnah11*^*iv/iv*^ mutants) and targeted mutation of the putative flow sensor (*Pkd1l1*^*tm1/tm1*^ mutants) would result in the same phenotype. Indeed, at E13.5, the L-R defects of *Dnah11*^*iv/iv*^ and *Pkd1l1*^*tm1/tm1*^ embryos are highly similar (Fig 1C, 1F and S2E Fig). In wild-type embryos, *Nodal* is expressed in the left LPM for around 6–8 hours beginning at the 3 ss [38, 39]. However, we found that the timing of LPM Nodal cascade induction, assessed by WISH for *Pitx2*, was subtly different between *Dnah11*^*iv/iv*^ and *Pkd1l1*^*tm1/tm1*^ mutants. While *Pitx2* expression was evident at the 3 ss but not before in control embryos, loss of flow in *Dnah11*^*iv/iv*^ mutants delayed the onset of *Pitx2* until the 4 ss (Fig 2E); a delay of around 2 hours, perhaps resulting from a slower and stochastic activation of the cascade in the absence of the flow signal [35]. No such delay was evident in *Pkd1l1*^*tm1/tm1*^ mutants; 100% of 3 ss *Pkd1l1*^*tm1/tm1*^ embryos exhibited LPM *Pitx2* expression, and a single embryo displayed expression at the 2 ss (Fig 2E). Coupled to the above findings, this favors a model wherein nodal flow drives the timely repression of *Pkd1l1* on the left side: when *Pkd1l1* function is perturbed in *Pkd1l1*^*tm1/tm1*^ mutants, the Nodal cascade activates bilaterally without delay.

### *Pkd1l1* Acts Upstream of *Cerl2* Asymmetry during Left-Right Development

**Fig 3:**
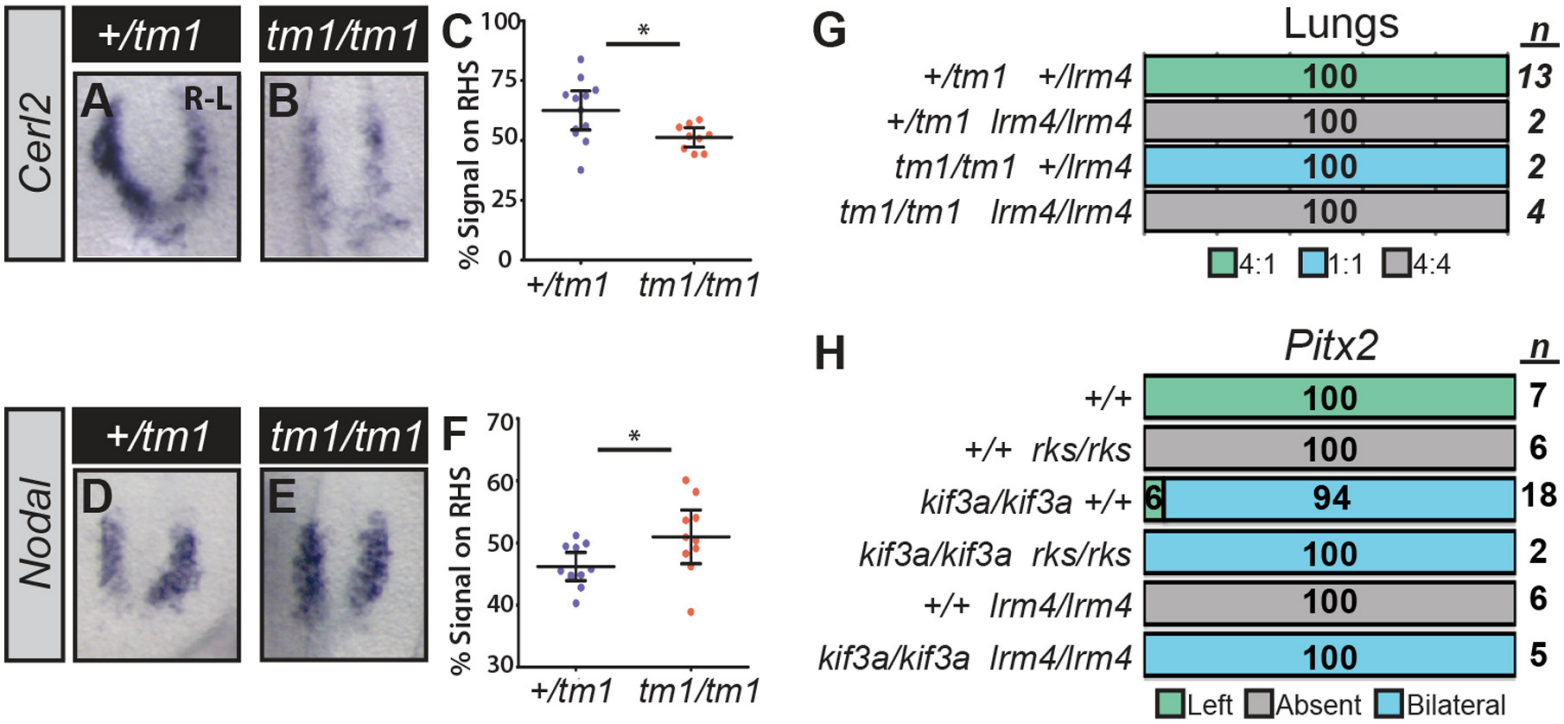
The Genetic Relationship between *Pkd1l1*, *Pkd2*, and Cilia. (A-F) *Cerl2* (*A-C*) and *Nodal* (*D-F*) expression at the node of *Pkd1l1*^*tm1/tm1*^ and control embryos. Quantitation of *in situ* signal reveals expression of both genes to be more symmetrical in mutant embryos (*C*, *F*). *—p<0.05, unpaired *t*-test applied. Error bars represent 95% confidence intervals. (G-H) Lung situs *(G)* (assessed at E13.5) and *Pitx2* expression *(H)* (assessed at E8.5) for embryos of the indicated genotypes, with the percentage of embryos exhibiting each phenotype and the total number given.

At the LHF to early somite stages, the action of nodal flow represses *Cerl2* in left-sided node crown cells, resulting in the R>L *Cerl2* expression bias [8, 40, 41]. We therefore assessed *Cerl2* asymmetry in *Pkd1l1*^*tm1/tm1*^ mutants by WISH; we quantified *in situ* staining and then calculated the percentage of total stain that was present in right-sided crown cells. In order to control for observation bias, samples were processed together, scored for L-R expression and genotyped only after analysis. As expected, the percentage of *Cerl2* stain was greater on the right side than the left side in control embryos measured at the 1–3 ss when *Cerl2* asymmetry was obvious by inspection (Fig 3A and 3C). In contrast, *Cerl2* asymmetry failed to manifest in *Pkd1l1*^*tm1/tm1*^ mutants, which instead exhibited a symmetrical pattern of *Cerl2* expression (Fig 3B and 3C). Consistent with this, most *Pkd1l1*^*tm1/tm1*^ mutants exhibited symmetrical crown cell *Nodal* expression whereas *Nodal* showed the expected subtle R<L bias in control embryos (Fig 3D–3F). Thus, functional *Pkd1l1* is not required for crown cell *Cerl2* or *Nodal* expression *per se*, but it is needed for asymmetries in their expression to manifest downstream of nodal flow.

Close examination of stage-matched *Pkd1l1*^*tm1/tm1*^ and control embryos stained for *Cerl2* under identical experimental conditions, revealed that the bilaterally symmetrical expression in mutants represents bilateral downregulation of *Cerl2*, rather than the left-side only downregulation observed in wild-type embryos (Fig 3A and 3B). All samples were scored before genotyping, in order to remove observer bias. We furthermore repeated this staining then genotyping procedure multiple times and obtained the same results from different litters, supporting the conclusions (S5 Fig). Thus, genetically, *Pkd1l1* normally acts to maintain high levels of *Cerl2*. In contrast, *Cerl2* levels remain bilaterally high (derepressed) in *Pkd1l1*^*rks/rks*^ and *Pkd2*^*lrm4/lrm4*^ embryos [16]; these differences in crown cell *Cerl2* levels between *Pkd1l1*^*tm1/tm1*^ and *Pkd1l1*^*rks/rks*^ embryos would be expected given the distinct LPM Nodal cascade activities found in these mutants.

### The Genetic Relationship between *Pkd1l1*^*tm1*^ and *Pkd2*^*lrm4*^

Since it has been hypothesized that PKD1L1 and PKD2 act as a flow-sensing complex, it is somewhat surprising that opposite phenotypes are present in *Pkd1l1*^*tm1/tm1*^ (bilateral Nodal cascade) and *Pkd2*^*lrm4/lrm4*^ (absent Nodal cascade) mutants. To understand the genetic relationship between the two, we generated *Pkd1l1*^*tm1/tm1*^;*Pkd2*^*lrm4/lrm4*^ double mutant embryos. At E13.5, all *Pkd1l1*^*tm1/tm1*^;*Pkd2*^*lrm4/lrm4*^ double mutants exhibited right lung isomerism (Fig 3G and S2 Table), thereby phenocopying *Pkd2*^*lrm4/lrm4*^ single mutants and not *Pkd1l1*^*tm1/tm1*^ embryos (Figs 1F, 3G and S2 Table). This shows that *Pkd2* is genetically epistatic to *Pkd1l1*.

The data described so far suggest a genetic pathway linking nodal flow to the emergence of gene asymmetries within crown cells in which flow impacts *Pkd1l1* which lies genetically upstream of *Pkd2*. *Pkd2* activity, in turn, influences crown cell *Cerl2* and *Nodal* expression and, ultimately, activation of the LPM Nodal cascade. In depth discussion of this genetic scheme and further interpretation of these results are included in the **Discussion**.

### *Pkd1l1* and *Pkd2* Require Cilia to Function

Immotile cilia found on crown cells are required for the sensation of nodal flow [42]. Since PKD1L1 and PKD2 localize to nodal cilia [17, 18], we asked whether cilia were required for *Pkd1l1* and/or *Pkd2* function *in vivo*. Loss of the anterograde intraflagellar transport motor component, KIF3A, results in a failure of ciliogenesis and bilateral induction of the LPM Nodal cascade (Fig 3H) [34, 43]. Both *Pkd1l1*^*rks/rks*^;*Kif3a*^*–/–*^ and *Pkd2*^*lrm4/lrm4*^;*Kif3a*^*–/–*^ double mutants exhibited bilateral *Pitx2* expression, the phenotype of *Kif3a*^*–/–*^ single mutants, whereas *Pkd1l1*^*rks/rks*^ and *Pkd2*^*lrm4/lrm4*^ single mutants lacked LPM *Pitx2* expression (Fig 3H and S3 Table). The simplest explanation of this phenotype, that the *Pkd1l1*^*rks/rks*^ and *Pkd2*^*lrm4/lrm4*^ phenotypes can be suppressed by loss of cilia, suggests that *Pkd1l1* and *Pkd2* require cilia to function *in vivo*. This genetic evidence is backed up by the findings that PKD2 and PKD1L1 localize to cilia [16–18].

### The *Pkd1l1*^*rks*^ Mutation Destabilizes an Extracellular PKD Domain

**Fig 4:**
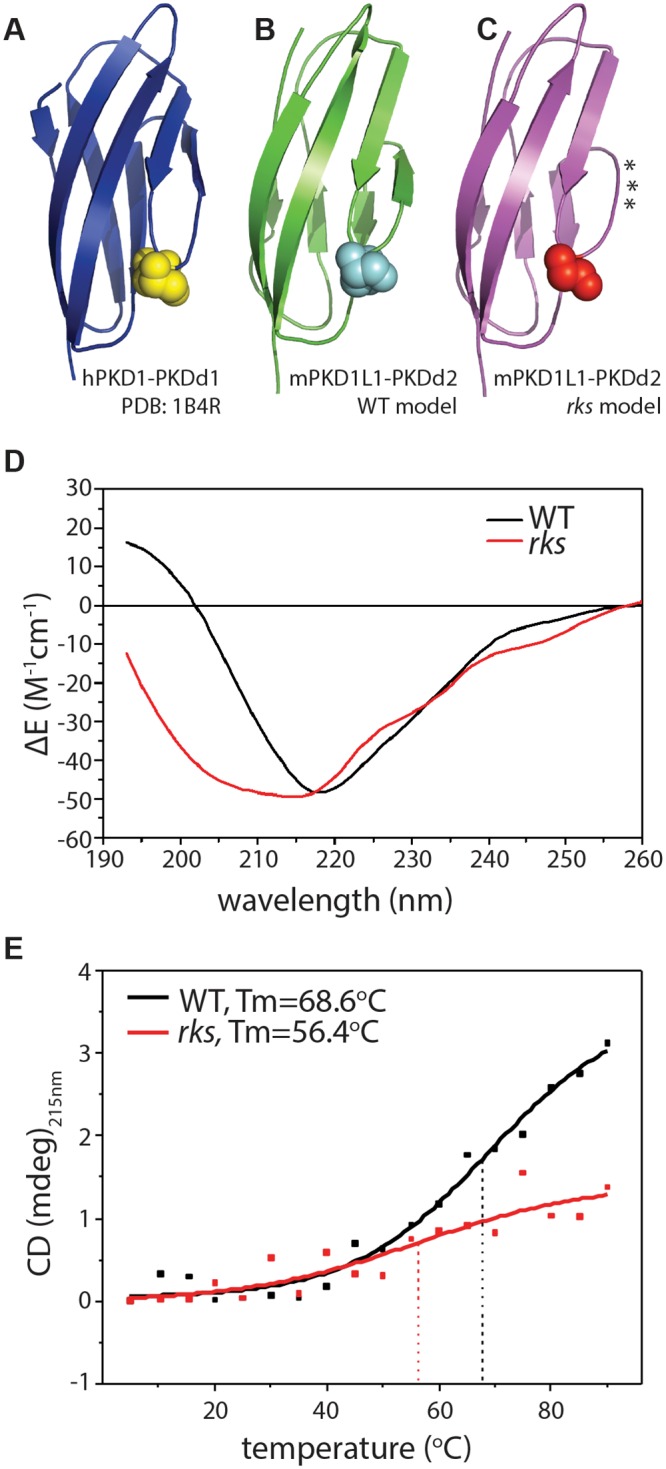
Destabilization of a PKD Domain by the *Pkd1l1*^*rks*^ Mutation. (A-C) Structure of human PKD1 PKD domain 1 (*A*) and models of mouse PKD1L1 PKD domain 2; wild-type (*B*) or *rks*-mutated (*C*). Domains are largely composed of β-sheets (block arrows). The aspartic acid mutated in *Pkd1l1*^*rks*^, or its equivalent in PKD1, is shown in space-fill. The asterisks denote loss of secondary structure in the *rks*-mutated domain. (D) SRCD spectroscopy of mouse PKD1L1 PKD domain 2 for wild-type and *rks*-mutated domains. Spectra are consistent with decreased stability (decreased secondary structure) in mutated domains. (E) Thermal denaturation analysis of PKD1L1 PKD domain 2: a reduced melting temperature (Tm) of 56.4°C is evident in the *rks*-mutated domain; in wild-type controls a Tm of 68.6°C is detected.

We next addressed how the *Pkd1l1*^*rks*^ mutation impacts PKD1L1 protein function. Our genetic experiments comparing the targeted allele *Pkd1l1*^*tm1*^ to *Pkd1l1*^*rks*^ had suggested that *Pkd1l1*^*rks*^ is a non-null allele (Fig 1F and 1M). The *Pkd1l1*^*rks*^ point mutation itself resides in the second of two polycystic kidney disease (PKD) domains within the extracellular N-terminal portion of the protein (Fig 1A) [16]. An NMR structure of the first PKD domain from human PKD1, a close homolog of PKD1L1, revealed the domain to be a ß-sandwich fold consisting of seven ß-strands (Fig 4A) [44]. By extending our previous fold recognition modeling approaches [16], we have predicted the second PKD domain of mouse PKD1L1 to have a similar fold topology (Fig 4B). To validate our model, we performed structural studies on PKD1L1 by synchrotron radiation circular dichroism (SRCD) spectroscopy [45]. Conformational studies in solution of the purified second PKD domain from mouse PKD1L1 showed the typical appearance of a ß-strand protein (Fig 4D) consisting of 44% ß-strand and 56% disordered (linkers and loops between strands) by Raussens algorithm [46], in agreement with our molecular model.

We then assessed the impact of the *Pkd1l1*^*rks*^ point mutation upon the structure of PKD1L1’s second PKD domain. Solution studies by SRCD of *rks*-mutated purified PKD domains shifted the SRCD spectra, which now exhibited characteristics of increased disorder (Fig 4D), with structural content changed to being 31% ß-strand and 69% disordered. Thermal denaturation studies on both wild-type and *rks*-mutated PKD domains showed the wild-type domain to have a higher melting temperature (Tm) of 68.6°C, compared to 56.4°C for the *rks*-mutated domain (Fig 4E). Both of these lines of evidence support the notion that the *Pkd1l1*^*rks*^ point mutation destabilizes the extracellular PKD domain. Indeed, such a destabilization was also observed in our molecular models which predicted an increase in disorder reflected by a loss of secondary structure in the C’ ß-strand in particular, marked by asterisks in Fig 4C (see [44] for PKD domain ß-strand nomenclature).

In *Pkd1l1*^*rks/rks*^ mutants, it is possible that the point mutation causes a drastic inability of the protein to properly fold or be localized to the correct cellular compartment. Whilst we cannot fully rule out this possibility, it seems unlikely since the phenotype of *Pkd1l1*^*rks/rks*^ mutants is very different to *Pkd1l1*^*tm1/tm1*^. Moreover, *Pkd1l1*^*rks*^ is dominant over *Pkd1l1*^*tm1*^ whilst neither *Pkd1l1*^*rks*^ nor *Pkd1l1*^*tm1*^ exhibit phenotypes in the heterozygous state. Thus, we favour a model in which the PKD domain structural destabilization we have observed results in abrogated functionality of that domain within the protein, thereby implicating the PKD domain in PKD1L1 function in L-R patterning. It is worth noting that similar point mutations in PKD domains of PKD1 also impact the stability and mechanical properties of PKD domains [47].

### PKD1L1 Mediates a Response to Artificial Fluid Flow

After establishing that *Pkd1l1* is necessary for L-R patterning, we then sought to test sufficiency of PKD1L1 in the response to flow in a context in which we can control fluid flow and assay the cells Ca^2+^ signal response. We therefore addressed this issue by asking the question: can PKD1L1 function in the cellular response to shear stress induced by fluid flow?

**Fig 5:**
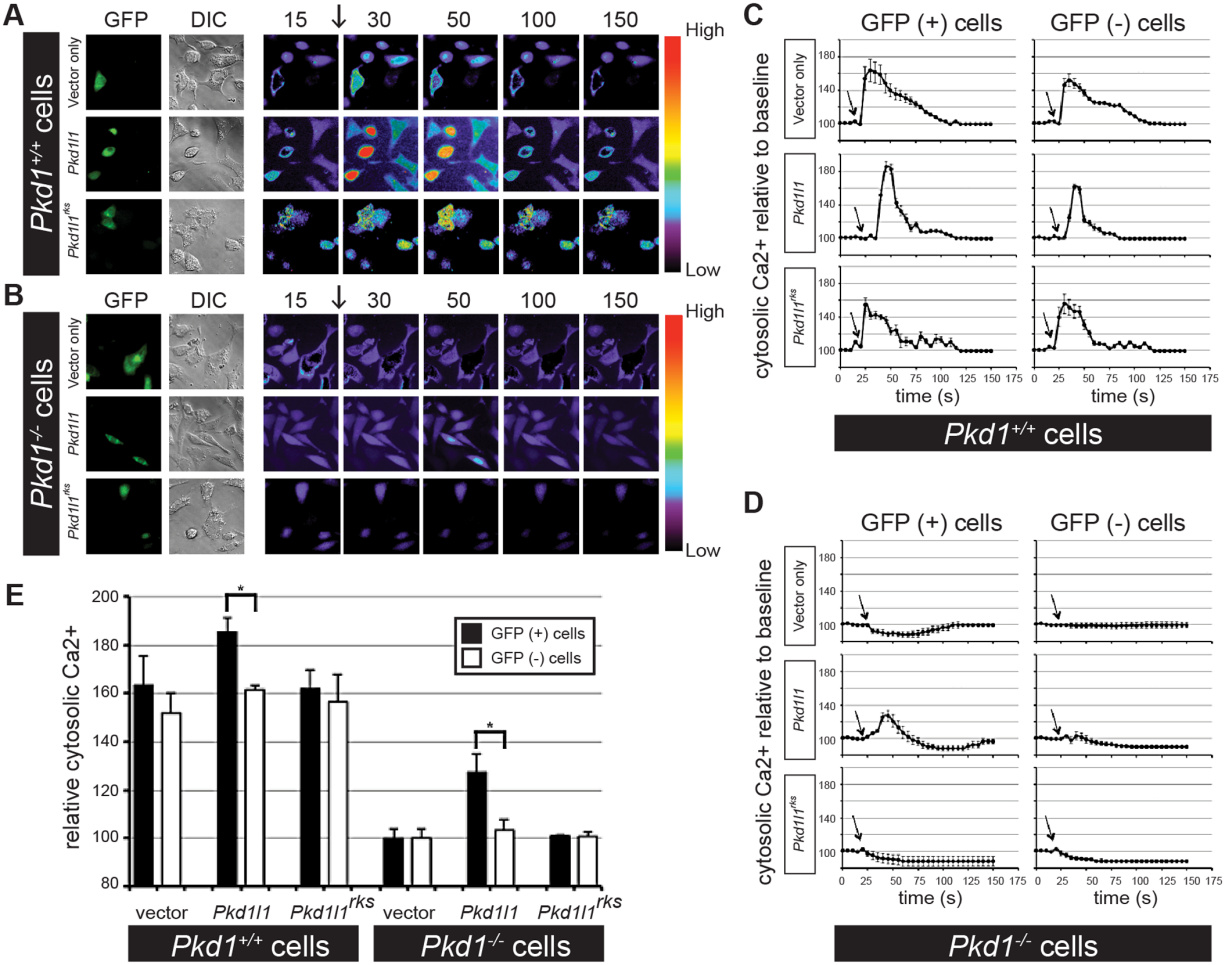
Flow-Induced Ca^2+^ Signaling Depends on PKD1L1. (A-B) *Pkd1*^*+/+*^ *(A)* and *Pkd1*^*–/–*^*(B)* cells were transfected with vector-GFP alone, PKD1L1-GFP or PKD1L1^rks^-GFP. Successfully transfected cells had green fluorescence (GFP), and the entire cell population was observed by DIC. After baseline Ca^2+^ level was taken, fluid-shear stress was applied to cells (arrow). Numbers indicate time in seconds (s). Color bars indicate Ca^2+^ level (pseudocoloured), where black-purple and yellow-red represent low and high Ca^2+^ levels, respectively (C-D) Quantitation from independent experiments of *Pkd1*^*+/+*^ *(C)* and *Pkd1*^*–/–*^*(D)* cells was averaged and plotted in line graphs. Within the same cell population, successfully transfected (GFP+) and non-transfected (GFP-) cells were analyzed separately. Arrows indicate the start of fluid-shear stress. Time is indicated in seconds (s). (E) Statistical analysis was done by analyzing the peak changes of intracellular Ca^2+^. While vector-GFP is used as a negative control, non-transfected cells (GFP-) were also used as an internal control. n = 150 cells for each group in three independent transfections. *—p<0.05.

In vascular endothelial cells and kidney epithelial cells, flow-induced shear stress elicits a Ca^2+^ signaling response. This signaling event depends on primary cilia as well as the Polycystin protein PKD1; cells isolated from *Pkd1*^*–/–*^ mice do not undergo flow-induced Ca^2+^ signaling (FICS) [48, 49]. We utilized this experimentally tractable system to ask how expression of PKD1L1 impacted FICS. We loaded ciliated endothelial cells with the Ca^2+^-binding dye Fura2-AM, applied fluid flow at 7.2 dyne/cm^2^, then recorded Ca^2+^ response by ratiometric fluorescence imaging. In Fig 5A and 5B we show representative images from selected time-points, whilst in Fig 5C and 5D we show averaged traces from multiple fields of view for the changes in intracellular Ca^2+^ after the onset of flow (arrows). Finally, the results of three independent transfections, with quantitation of 50 GFP+ and 50 GFP- cells per transfection, are collated in Fig 5E (see S5 Table for numerical results); statistical comparison among groups was performed using ANOVA followed by Tukey’s posttest, with *p*<0.05 taken as statistically significant differences. In wild-type cells, a transient Ca^2+^ response upon the onset of fluid flow was observed (Fig 5A, 5C and 5E). As anticipated, loss of PKD1 in cells generated from *Pkd1*^*–/–*^ mice abolished the FICS response (Fig 5B, 5D and 5E). Expression of a PKD1L1-GFP construct was able to restore FICS in *Pkd1*^*–/–*^ cells and to increase the magnitude of the response in *Pkd1*^*+/+*^ cells (Fig 5A–5E). Importantly, we did not see a restoration in either untransfected cells (GFP-negative) within the same sample or within separate samples that were transfected with GFP alone (Fig 5A–5E). These experiments demonstrate that PKD1L1 can mediate a Ca^2+^ response to fluid flow. In contrast to wild-type PKD1L1-GFP, expression of PKD1L1^rks^-GFP protein did not rescue FICS in *Pkd1*^*–/–*^ cells (Fig 5A–5E). Thus, in our cell line system, PKD1L1 restores FICS in the absence of PKD1. In the node, *Pkd1l1* acts downstream of flow and upstream of *Pkd2* and *Cerl2/Nodal* asymmetries in the sensation pathway.

### Proper Localization of PKD2 to Nodal Cilia Requires PKD1L1

Finally, we turned our attention to the localization of PKD2 protein in the node. In contexts where PKD1 and PKD2 function together, PKD2 localization to cilia has been shown to depend on the presence of PKD1 [48]; this perhaps explains why PKD2 is not functional in the absence of PKD1 [50]. By contrast, we find bilateral *Nodal* expression in *Pkd1l1*^*tm1/tm1*^ mutants, implying bilateral PKD2 activity in this *Pkd1l1* mutant background. Two possibilities that could explain this apparent contradiction are: (1) PKD2 localization to cilia is not required for its role in L-R patterning; or (2) PKD2 can still localize to cilia and signal in the *Pkd1l1*^*tm1/tm1*^ mutant. We tested these by assessing PKD2 subcellular localization within the node of wild-type and mutant embryos at 8.0 dpc.

**Fig 6:**
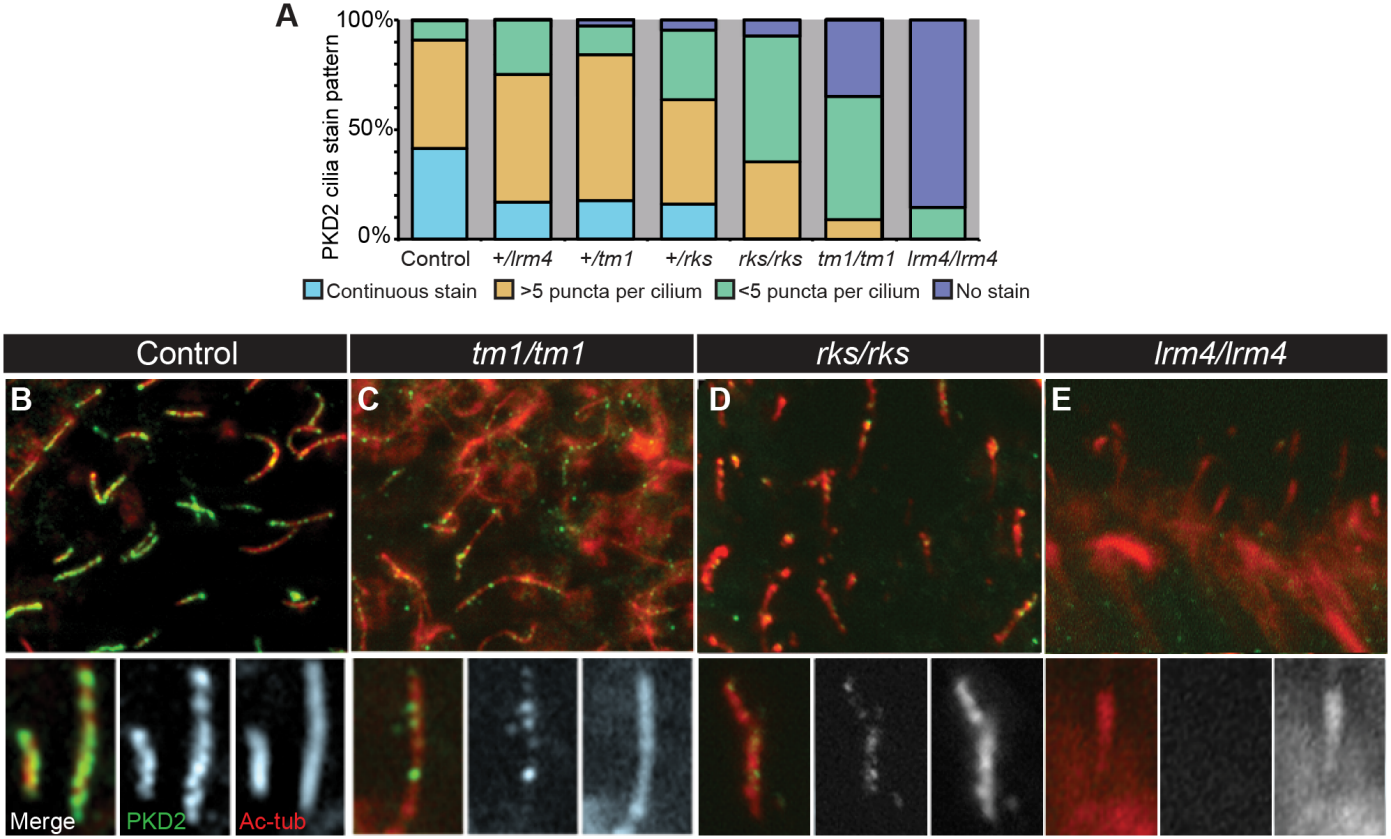
Cilia and PKD2 Localization and Function. (A-E) PKD2 localization in nodal cilia of embryos of the indicated genotype. Staining was divided into categories and quantitation is given in (*A*). In *(A)*, all genotypes are statistically significantly different from each other (p<0.001) except for *Pkd2*^*+/lrm4*^ and *Pkd1l1*^*+/tm1*^ which are statistically not significantly different.

In wild-type control embryos, PKD2 localized to all nodal cilia (Fig 6A and 6B), as previously reported [18]. Most cilia showed continuous PKD2 staining along the ciliary axoneme, marked by acetylated α-tubulin, or greater than five PKD2 puncta per cilium (Fig 6A and 6B). By contrast, PKD2 was absent from the vast majority of cilia in *Pkd2*^*lrm4/lrm4*^ mutants (Fig 6A and 6E), in agreement with the finding that the *lrm4* mutation prevents a PKD2-Venus fusion from localizing to nodal cilia in transient transgenic mouse embryos [51]. The *lrm4* point mutation changes a residue in the first extracellular loop of PKD2 (Fig 1A) [32], but does not affect its channel activity [51]. It is currently not known why PKD2^lrm4^ protein fails to properly localize to nodal cilia. Nevertheless, given the striking L-R defects of *Pkd2*^*lrm4/lrm4*^ mutants, these data suggest a requirement for PKD2 protein localization to nodal cilia; without PKD2 in cilia, the downstream Nodal cascade is not activated on either the left or right sides, resulting in right isomerism in mutants.

In *Pkd1l1*^*tm1/tm1*^ mutants, we observed reduced PKD2 ciliary levels compared to controls (Fig 6A and 6C), showing that proper levels of functional PKD1L1 are needed for the efficient import or retention of PKD2 in nodal cilia. *Pkd1l1*^*+/tm1*^ heterozygous embryos exhibited reduced ciliary PKD2 (Fig 6A), suggesting that PKD2 levels within nodal cilia are controlled, at least in part, by the dosage of PKD1L1. Thus, whilst we do observe reduced PKD2 in the nodal cilia of *Pkd1l1*^*tm1/tm1*^ mutants, this is not a complete loss of PKD2 as has been observed in distinct cell types when *Pkd1* is ablated [48]. An assessment of PKD1L1 localization and levels in these mutant backgrounds will require functional PKD1L1 antibodies, which are currently unavailable. Finally, we also assessed the PKD2 localization to nodal cilia in *Pkd1l1*^*rks/rks*^ mutants and found that it was also reduced (albeit to a lesser extent than in *Pkd1l1*^*tm1/tm1*^) in *Pkd1l1*^*rks/rks*^ mutants (Fig 6A and 6D). Together, these data suggest that PKD2 localization to cilia does not depend entirely on functional PKD1L1.

## Discussion

Addressing how nodal flow is sensed by the embryo to elicit downstream asymmetries in gene expression is critical if we are to understand the early phases of L-R patterning. We, and others, have previously implicated the Polycystin proteins PKD1L1 and PKD2 in the sensation of nodal flow. Here, we reveal that *Pkd1l1* acts in a genetic pathway at the level of flow sensation, placing *Pkd1l1* function between flow and *Pkd2*. Moreover, we demonstrate that PKD1L1 protein is sufficient to elicit a Ca^2+^ signal in response to artificial fluid flow.

### A Genetic Repression Model of Nodal Flow Sensation

Our genetic experiments suggest a pathway in which flow acts upstream of *Pkd1l1* which, via *Pkd2* (that lies downstream on the pathway), signals to affect crown cell *Cerl2* and *Nodal* levels. The result of this pathway, initiated by nodal flow, is to increase left-sided crown cell NODAL signalling and thereby activate the Nodal pathway in the left LPM only.

**Fig 7:**
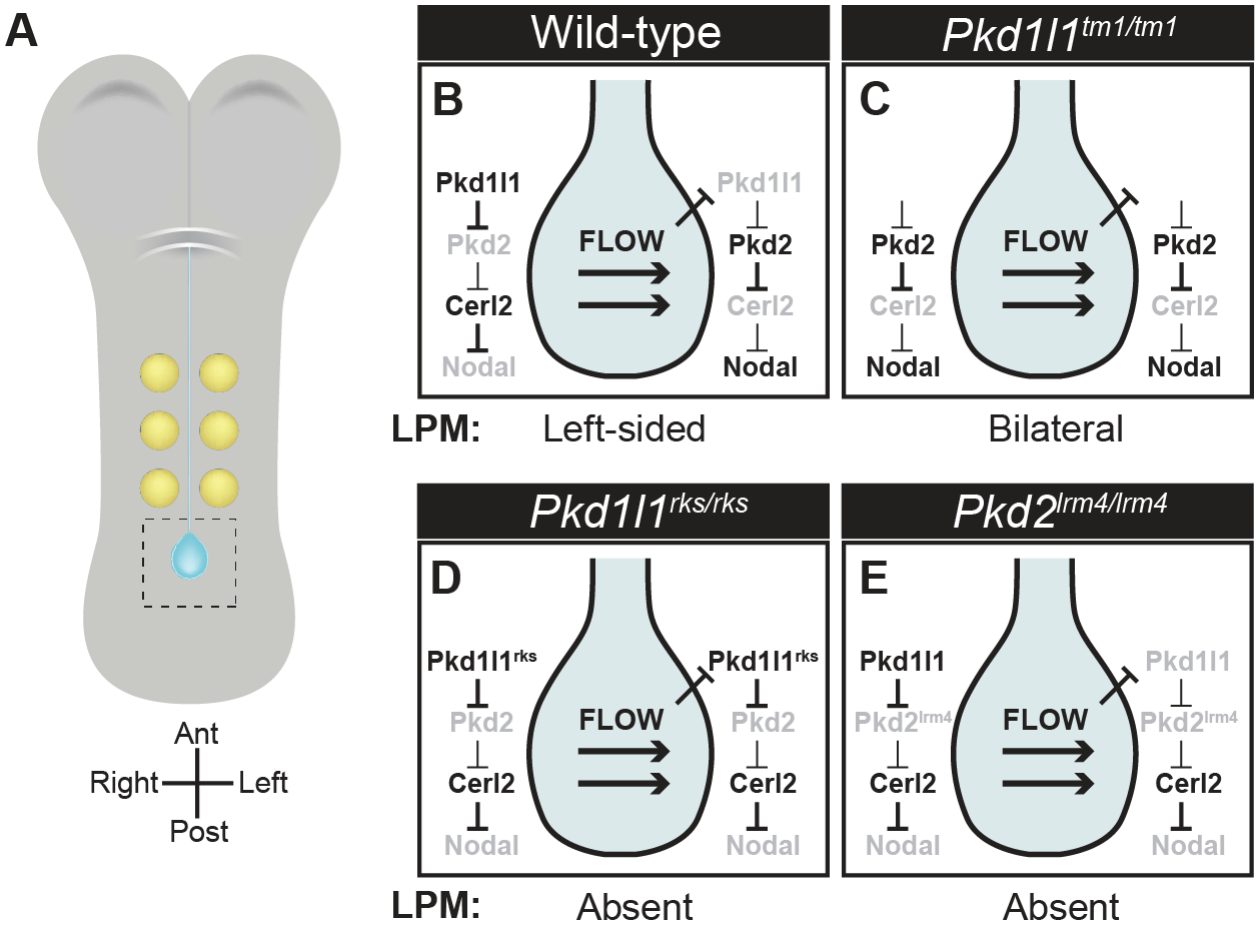
Multi-repression Model for L-R Asymmetry Determination in Crown Cells. (A) Schematic of a 3 ss flat-mounted mouse embryo showing somites (yellow), and node (blue). (B) Pictorial representation of the multi-repression model in which flow represses *Pkd1l1* on the left side, resulting in the derepression of *Pkd2*, inhibition of *Cerl2* and, as a result, higher NODAL activity on the left. (C-E) Predictions of the multi-repression model in various genetic mutants including the impact on the crown cell genetic pathway as well as the predicted LPM Nodal cascade activity.

We can further extend our genetic results to interpret the nature of the interactions between adjacent genes on the pathway (summarized in Fig 7). Since the *Pkd1l1*^*tm1/tm1*^ is likely to be hypomorphic or null (S1 Fig) i.e. loss-of-function, and *Pkd1l1*^*tm1/tm1*^ mutants exhibit bilateral LPM Nodal cascade activity, we suggest that *Pkd1l1* normally represses Nodal cascade activation and that this repression must be relieved by nodal flow on the left side only. Moreover, since *Pkd1l1*^*tm1/tm1*^ and *Pkd2*^*lrm4/lrm4*^ mutants have opposite crown cell *Cerl2* and *Nodal* phenotypes as well as distinct LPM *Nodal* phenotypes, and that the *Pkd2*^*lrm4/lrm4*^ phenotype manifests in double mutants, we suggest that, genetically, *Pkd1l1* acts as an upstream repressor of *Pkd2*. Finally, *Pkd2* is known to act as a genetic repressor of *Cerl2* which itself is an antagonist of the Nodal pathway. This model, described pictorially in Fig 7A and 7B, coheres with the genetic tests we have performed in this study. It is worth noting that this interpretation of the data relies on *Pkd1l1*^*tm1*^ being a hypomorphic or null allele, something which is very likely (S1 Fig), but owing to the absence of functional PKD1L1 antibodies, not something we have formally tested at the protein level. It is formally possible that a cryptic initiation codon downstream of the deleted exons in *Pkd1l1*^*tm1*^ results in a portion of PKD1L1 being expressed in mutants, and our repression model must be considered with this caveat in mind. Nevertheless, the model predicts bilateral Nodal cascade activity in the absence of *Pkd1l1* (Fig 7C), something we do see in *Pkd1l1*^*tm1/tm1*^ mutants, as well as absent Nodal cascade activity in loss-of-function *Pkd2* mutants like *Pkd2*^*lrm4/lrm4*^ (Fig 7E), again in agreement with experiment. Moreover, it is worth emphasizing that this is a genetic model and that our study leaves open several possibilities regarding the nature of the molecular interactions between adjacent members of this genetic cascade. The genetic relationships uncovered here could manifest via direct molecular repression of PKD2 by PKD1L1 or more indirectly by the control of PKD1L1 and/or PKD2 localization.

Our multi-repression-based genetic model ascribes two functions to *Pkd1l1* in L-R patterning: (1) to mediate a flow-based signal (or, genetically, to receive a signal from *Dnahc11*, mutants of which we use in our genetic experiments to eliminate flow) and (2) to genetically repress *Pkd2* activity. In the absence of flow, *Pkd1l1* represses *Pkd2* in the node. The onset of flow (*Dnahc11* function) relieves this repression, causing *Pkd2* to activate and thereby initiates a cascade that results in left-sided Nodal signals. In the absence of *Pkd1l1*, repression of *Pkd2* is relieved and signals are activated bilaterally independently of flow (since the *Dnahc11*^*iv*^ mutation does not manifest in the presence of *Pkd1l1*^*rks*^). By contrast, since *Pkd2* is required for *Cerl2* repression and *Nodal* activation, loss of *Pkd2* function results in bilateral absence of the Nodal cascade. The repression model provides an explanation for the previously published *Pkd1l1*^*rks*^ mutant phenotype since the *Pkd1l1*^*rks*^ mutation seems to uncouple the two functions of *Pkd1l1*. The *Pkd1l1*^*rks*^ mutation structurally disrupts an extracellular PKD domain, resulting in PKD1L1^rks^ protein being unable to elicit Ca^2+^ signals downstream of artificial flow. Moreover, NODAL activity is bilaterally repressed in *Pkd1l1*^*rks/rks*^ mutants, implying that *Pkd2* is inactive regardless of the presence of normal nodal flow in these mutants. This is consistent with the idea that the *Pkd1l1*^*rks*^ mutation is loss-of-function with respect to role (1), flow sensation, but, owing to its insensitivity to flow, it appears gain-of-function with respect to role (2), *Pkd2* inhibition (i.e. *Pkd2* remains inhibited regardless of the presence of the upstream activating signal, nodal flow) (Fig 7D).

Furthermore, the repression model coheres with the subtle phenotypic differences between *Pkd1l1*^*tm1/tm1*^ and *Dnah11*^*iv/iv*^ mutants. Though these two mutants exhibit overtly similar phenotypes, with high levels of left lung isomerism and bilateral Nodal cascade activity, it is noteworthy that these outcomes result from distinct mechanisms. In *Dnah11*^*iv/iv*^ mutants, loss of flow results in stochastic (unbiased) Nodal pathway activation; crucially, initiation of the Nodal cascade in the LPM occurs with a delay of around 2 hours in the absence of flow. This is consistent with the repression model, since the pathway to activate Nodal robustly requires repression of *Pkd1l1* by flow; this would not occur in a timely fashion in the absence of flow. In stark contrast, the *Pkd1l1*^*tm1*^ allele leads to bilateral LPM Nodal pathway activity without delay, interpreted in our model as loss of *Pkd1l1* leading to the fast derepression of *Nodal*. Why the Nodal cascade activates at all in the absence of flow is an intriguing question and suggests that repression of *Pkd1l1* by flow is not absolutely required for *Nodal* activation but, rather, the repression is required for biased expression to manifest at the correct time. If this pathway is not activated in this way owing to lack of flow, stochastic changes in fluid movement, or other variations, followed by positive feedback loops operating at the node and LPM [14, 40] likely result in randomized or bilateral Nodal activity.

### *Pkd1l1*, *Pkd2*, and Cilia

Our experiments, in which we generated *Pkd1l1*^*rks/rks*^;*Kif3a*^*-/-*^ and *Pkd2*^*lrm4/lrm4*^;*Kif3a*^*-/-*^ double mutants and found that they exhibited the same LPM Nodal pathway activity as *Kif3a*^*-/-*^ mutants, show that cilia (or, strictly, *Kif3a*) are required for the manifestation of the *Pkd1l1* and *Pkd2* mutant phenotypes. A simple explanation of this finding is that PKD1L1 and PKD2 require cilia to function, in agreement with their sub-cellular localization to cilia [16–18, 42, 52]. However, this relatively complicated genetic interaction warrants further discussion. In *Kif3a*^*-/-*^ mutants, the vast majority of nodal cilia are lost [34, 43] and the Nodal cascade is bilaterally activated. This suggests that cilia provide an inhibitory signal preventing LPM Nodal cascade activation. In contrast, loss of *Pkd2* results in the absence of LPM *Nodal*, implying that *Pkd2* acts to overcome this inhibition. In *Pkd2*^*lrm4/lrm4*^;*Kif3a*^*-/-*^ double mutants, the loss of cilia-mediated inhibition results in *Nodal* being activated (now bilaterally) independently of *Pkd2*. Therefore, loss of that cilia-based inhibition (in *Kif3a*^*-/-*^) suppresses loss of *Pkd2*. However, since *Pkd1l1*^*tm1*^ does not suppress *Pkd2*^*lrm4*^, it seems likely that the cilia-based inhibitory signal is not provided by *Pkd1l1*. These data speak to an apparent paradox in the field; since PKD2 acts to activate LPM *Nodal* and is thought to act in cilia, how can loss of cilia result in bilateral Nodal activity? One possibility is that Hedgehog signaling, which acts in the LPM bilaterally upstream of Nodal activation [53], could be augmented sufficiently to affect *Nodal* expression upon loss of cilia. Moreover, loss of *Kif3a* impacts both flow generation and sensation in the node [5, 18] and also affects midline structures resulting in barrier defects. Thus, the inhibitory signal (in terms of Nodal activation) provided by cilia independently of *Pkd1l1/Pkd2* function could be acting at the node, the LPM, the midline, or in a combination of these locations. However, the most important contribution likely comes from crown cell cilia since embryos expressing *Kif3a* solely in crown cells express *Nodal* exclusively in the left LPM following application of leftward flow [42]. This result coheres with our interpretation of the *Pkd1l1*^*rks/rks*^;*Kif3a*^*-/-*^ and *Pkd2*^*lrm4/lrm4*^;*Kif3a*^*-/-*^ double mutant phenotypes because the cilia-based repressive signal, that cannot be overcome by mutation of *Pkd1l1* or *Pkd2*, appears to be acting most dominantly within the sensory cilia of the crown cells.

The finding that loss of cilia masks the *Pkd2*^*lrm4*^ mutant phenotype reinforces the idea that PKD2 must localize to nodal cilia to activate downstream Nodal signals [51], (and this manuscript). Others have demonstrated a requirement for PKD1 or PKD1L1 in the localization of PKD2 to cilia [16, 17, 48]. If PKD2 localization to cilia were entirely dependent on wild-type PKD1L1, we might expect no PKD2-mediated Nodal cascade activation in *Pkd1l1*^*tm1/tm1*^ mutants, the opposite of the bilateral Nodal activity that we do see. However, our results show this clearly not to be the case; some PKD2 still localizes to nodal cilia in *Pkd1l1*^*tm1/tm1*^ mutants. Although there is less PKD2 in the nodal cilia of *Pkd1l1*^*tm1/tm1*^ mutants compared to wild-type, interpreting this result in light of our genetic model suggests this lower amount of PKD2 to be active in the absence of PKD1L1. These findings further suggest that PKD2 cannot respond to nodal flow in the *Pkd1l1*^*tm1/tm1*^ mutant since in these mutants nodal flow is normal and some PKD2 localizes to cilia; however, we see no evidence for consistent lateralized downstream Nodal cascade activity, implying that no flow sensation is occurring.

### PKD1L1 and the Response to Nodal Flow

Whether PKD1L1 is part of a pathway mediating sensation of an asymmetrically distributed chemical determinant positioned by nodal flow (chemosensation), or the force of flow itself (mechanosensation), remains a topic of debate [16, 22, 42, 54]. Here, we demonstrate that, in a tissue culture system where the onset of flow and the presence/absence of PKD1L1 is controlled, PKD1L1 cell autonomously mediates Ca^2+^ signaling downstream of fluid flow. Thus, our work favours a mechanosensation model of nodal flow sensation.

Left-biased Ca^2+^ signals have been observed around the node and thus correlate with left-sided Nodal activation [18, 36, 55]; these signals depend on both flow and PKD2. Thus, non-functional *Pkd2* alleles, such as *Pkd2*^*lrm4*^, should abolish this Ca^2+^ signal and lead to loss of *Nodal* induction, a result we find in *Pkd2*^*lrm4/lrm4*^ mutants. In *Pkd1l1*^*tm1/tm1*^ mutants, we observe bilateral *Nodal*, suggesting bilateral Ca^2+^ signaling. Extrapolation of our genetic model, in which *Pkd1l1*^*tm1/tm1*^ represents loss of *Pkd1l1*, into a simple molecular model can explain this: on the left side of the node, PKD2 repression by PKD1L1 is relieved by flow, resulting in the opening of PKD2 channels and activation of the Nodal cascade. In our tissue culture system, we do not see Ca^2+^ signals upon loss of the cells endogenous flow sensor, PKD1. Importantly, upon expression of PKD1L1 in this system, the cells again become competent to respond to flow, demonstrating that PKD1L1 can mediate flow sensation. PKD2 activity in *Pkd1l1*^*tm1/tm1*^ mutant embryos could not be recapitulated in the cell line we used because PKD1, not PKD1L1, is the endogenous flow sensor. Thus, the two systems provide complementary information, but are not directly analogous. Of note is that either PKD1 or PKD1L1 can function independently to mediate flow sensation in our tissue culture flow chamber system. The embryonic system provides us with a genetic model that can explain the mouse mutant phenotypes, whereas the tissue culture experiments reveal PKD1L1 protein to be able to mediate a cellular response to flow sensation.

With this in mind, though our data supports the mechanosensation model, this study does not rule out the importance of asymmetrically distributed molecules in the node cavity. Modeling studies demonstrate the viability of the concept of L-R asymmetric morphogen gradients within the node [56]. Nodal vesicular parcels (NVPs), large membrane-encased vesicles, have been described transiting the node and opening on the left side [23] and these might asymmetrically deliver a left-side determinant. Indeed, recently CERL2 protein has been demonstrated to transition from right-biased to left-biased in response to nodal flow; the timing of this event is notable in that left-biased localization is not detected until several hours after the establishment of L-R asymmetry at the node, when it functions to shut down NODAL activity and thereby lock in asymmetry [57]. Future study will determine whether flow-driven asymmetric distribution of molecules functions solely in this later event.

Nevertheless, other considerations favour the mechanosensation hypothesis. Firstly, the fact that the *Pkd1l1*^*rks*^ point mutation impacts one of two extracellular PKD domains implicates this domain in PKD1L1 function during L-R patterning. Interestingly, PKD domains are required in other contexts where mechanosensing occurs. For example, PKD1, which harbors fifteen PKD domains, is argued to act as a mechanosensor of fluid flow in the kidney [58]. However, this alone does not formally rule out a role for the PKD domain, or PKD1L1 in general, as a binding site for an unknown chemical determinant. Indeed, the Polycystin-1 family member PKD1L3 acts as a chemical receptor needed for sour taste perception [59], though it is noteworthy that PKD1L3 does not contain PKD domains.

Secondly, PKD domains exhibit remarkable mechanical strength along the N-to-C terminal axis [60, 61]. Some mutations in human PKD1 that cause ADPKD have been found within the PKD domains [47, 62]. An assessment of domain strength within these disease variants by atomic force microscopy found that many human disease associated point mutations caused a weakening of the PKD domains, suggesting that domain strength is critical for function [47]. Here, we found by molecular modeling, SRCD spectroscopy, and melting temperature analysis, that the *Pkd1l1*^*rks*^ point mutation structurally destabilizes the second PKD domain of PKD1L1. We thus hypothesize that such a destabilization leads to reduced domain strength and, therefore, the inability of PKD1L1^rks^ to transduce the force of nodal flow. This is corroborated by our finding that unlike PKD1L1, PKD1L1^rks^ protein does not mediate mechanosensation in response to fluid flow.

PKD1L1-PKD2L1 complexes in cilia of neural derived cells are known to facilitate a constitutive ciliary Ca^2+^ flux [52, 63], while a pressure clamp failed to induce signaling through PKD1L1-PKD2L1 complexes at physiological force levels. Whilst this somewhat contrasts with our data, it is worth noting that different cell types and a different PKD protein pair are examined in each study. Moreover, the nature of flow/force sensation by cilia will not necessarily translate to simple pressure upon the channel; cilia become deformed by motion within a flow leading to stresses along as well as perpendicular to the membrane. It is unclear to what extent all such forces will be modeled by pressure clamping.

The mechanistic details of how PKD1L1 mediates flow sensation in the node will require a multi-disciplinary approach combining structural biology, computational approaches, and genetics. Our finding that PKD1L1 and PKD2 function during L-R patterning requires primary cilia is intriguing; it has been argued that the bending of the ciliary membrane as a result of flow might be critical for mechanosensation by Polycystin proteins [48]. One possibility is that the N-terminus of PKD1L1 is tethered to the ciliary membrane so that PKD domains lie parallel to the membrane. Thus, when the membrane is bent by flow, the PKD domains would be placed under mechanical force along their N- to C-terminal axis, where they exhibit mechanical strength; this might aid in force transduction.

In summary, we reveal a genetic pathway for the early phases of L-R patterning which encompasses the generation of nodal flow, its’ sensation, and subsequent downstream changes in gene expression. Moreover, in a tissue culture system we demonstrate that PKD1L1 can mediate the sensation of fluid flow to elicit Ca^2+^ signals. Finally, we implicate a PKD domain of PKD1L1 in the response to flow and L-R patterning.

## Materials and Methods

### Ethics Statement

All experiments were performed under the guidelines and approval of the Home Office, UK and the MRC Harwell, UK Ethics Committee; Euthanasia was by cervical dislocation of adults and decapitation of embryos.

### Mice

The mouse lines used in this study are: *Pkd1l1*^*rks*^; *Pkd1l1*^*tm1Lex*^ (*Pkd1l1*^*tm1*^); *Pkd2*^*lrm4*^; *Dnah11*^*iv*^; *Kif3a*^*tm1Gsn*^ (*Kif3a*^*–*^); *Pkd1*^*tm1Jzh*^ (*Pkd1*^*–*^). All experiments were performed under the guidelines and approval of the Home Office, UK; Euthanasia was by cervical dislocation of adults and decapitation of embryos. *Pkd1l1*^*rks*^, *Pkd2*^*lrm4*^, and *Dnah11*^*iv*^ lines were congenic on C3H/HeH, while *Pkd1l1*^*tm1*^ and *Kif3a*^*–*^ were only used for experiments after at least four and two backcrosses to C3H/HeH, respectively.

### In Situ Hybridization

Whole mount in situ hybridization (WISH) was performed using digoxygenin-labeled riboprobes using standard procedures. Quantitation of crown cell gene expression was performed using ImageJ, NIH.

### Analysis of Embryonic Nodes

To visualize nodal cilia rotation, embryos were mounted on slides with the node facing up. Differential interference contrast (DIC) microscopy was then performed using a Leica DM2500 compound microscope with a monochrome high-speed Hamamatsu camera attached. Quantitation was performed by counting cilia rotations from movies taken at approximately 100 frames per second for at least 10 cilia per embryo and at least 3 embryos per genotype. For detection of nodal flow, fluorescent beads (0.2 μm; Invitrogen F-8848) were diluted 1:10 with dissection medium supplemented with 2% fetal bovine serum and then placed over mounted embryos. The node was imaged using a Zeiss Axio Observer Z1 microscope with a VivaTome extension to allow for high-speed optical sectioning. Particle Image Velocimetry (PIV) analysis was performed as previously described [37].

### Quantitative Reverse Transcription PCR (qRT-PCR)

RNA was extracted from single embryos using a Microplus kit (Qiagen) according to manufacturers’ instructions. 500 ng of RNA was used for cDNA synthesis using a SuperScriptIII First-Strand Synthesis SuperMix for qRT-PCR kit (Invitrogen). qPCR was performed in triplicate on a 7900 Fast Machine (Life Technologies) using Fast SYBR Green Mastermix (Life Technologies) with 20 ng cDNA and 500 nM forward and reverse primers in a final 20 μl reaction volume (see S4 Table for primer sequences). Quantitation was relative to *Hprt* and fold changes were calculated using the ΔΔC_T_ method (7500 Software v2.0.6., Life Technologies).

### Protein Expression and Purification

Both wild-type and *rks*-mutated versions of PKD domain 2 from mouse PKD1L1 were tagged at the N-terminus with His, expressed in *E*. *coli*, purified using a His column, refolded at decreasing urea concentration by affinity chromatography then vacufuged and dialysed. Protein concentration was calculated based on the measured ultraviolet (UV) absorbance (at 1 mm path length) at 280 nm on an Implen NanoPhotometer.

### *In Vitro* Flow-Induced Ca^2+^ Signaling (FICS) Assessment

Vascular endothelial cells were extracted from wild-type or *Pkd1*^*–/–*^ embryos as previously described [49]. Cells were cultured in permissive conditions (in the presence of interferon-γ at 33°C) to induce proliferation then transfected with 1 μg/mL pEGFP-N1 plasmid (Clontech Laboratories) containing EGFP only, PKD1L1-EGFP, or PKD1L1^rks^-EGFP. To promote cell differentiation and cilia growth, cells were grown under non-permissive conditions (in the absence of interferon-γ at 39°C) to induce differentiation.

For FICS experiments we used previously described protocols [64]. Briefly, cells were loaded with 5 μM Fura2-AM (Invitrogen) for 30 minutes at 39°C. Basal fluorescence was measured for one minute before the onset of fluid flow using a Nikon TE2000 microscope with Metafluor software. A shear stress of 7.2 dyne/cm^2^ was achieved in an FCS2 chamber with electrical enclosure heater (Biotechs, Inc.) at a flow rate of 550 μL/sec. Flow was then applied and fluorescence was monitored every 4 seconds. At the end of the experiment, minimum fluorescence measurements were obtained by treating cells with 2 mM EGTA and 10 μM ionomycin. After achieving the minimum signal, the maximum fluorescence was obtained by treating cells with excess calcium (10 mM). All fluorescence measurements were corrected for auto-fluorescence.

All quantifiable experimental values are expressed as mean +/- standard error of the mean (SEM), with values of p<0.05 being considered statistically significant. Data analysis was performed using SigmaPlot software Version 11 and comparisons among groups were done using ANOVA followed with Tukey’s posttest.

### Synchrotron Radiation Circular Dichroism (SRCD) Spectroscopy

Far UV SRCD experiments were performed using Module B of Beamline B23 at the Diamond Light Source (Didcot, Oxfordshire UK). Measurements of protein solutions were carried out in 20 mM Tris and 50 mM NaCl, pH8.5, at a concentration of 0.145 mg/ml (wild-type PKD domain) or 0.104 mg/ml (*rks*-mutated PKD domain). Four scans were taken at 20°C (1 nm increment, 1 s integration time and a scan rate of 38 nm/min) and averaged. Spectra of buffer alone were subtracted from the sample spectra. Secondary structure composition was calculated from the experimental spectra using Raussens algorithm [46].

## Supporting Information

S1 Fig Characterisation of the *Pkd1l1*^*tm1*^ allele. The *Pkd1l1*^*tm1*^ allele (*Pkd1l1*^*tm1Lex*^; [33]) comprises a beta-galactosidase-neomycin fusion gene (beta-geo) inserted in place of exons 3, 4 and 5 (labelled ‘LacZ’ in the schematic given in D). Importantly, this insertion contains a stop codon and polyA signal at its 3’ end. The data in this figure demonstrate that in *Pkd1l1*^*tm1/tm1*^ mutants, the gene is disrupted either by splicing onto the beta-geo cassette or by splicing around the cassette in a fashion that introduces a premature stop codon which truncates the protein very early, suggesting that *Pkd1l1*^*tm1*^ is a null or strong hypomorph. (AA’-AG’) Expression of *Pkd1l1* was assessed by LacZ staining, revealing that expression from the beta-geo reporter locus mirrors the endogenous expression pattern [16]. *Pkd1l1*^*+/tm1*^ embryos that were phenotypically normal; 7.5 dpc (AA’-AB’), 8.5 dpc (AC’-AE’), and 9.5 dpc (AF’-AG’) embryos were assessed. (B) WISH analysis of *Pkd1l1* expression in *Pkd1l1*^*+/tm1*^ and *Pkd1l1*^*tm1/tm1*^ embryos at 8.5 dpc. If all transcripts splice into beta-geo, then we would predict there to be no mRNA present for 3’ portions of *Pkd1l1*. However, WISH revealed equivalent expression patterns in both wild-type and mutant embryos (assessed with a probe covering exons 20–24); expression in the *Pkd1l1*^*tm1/tm1*^ embryos appears slightly reduced. (C) A proportion of *Pkd1l1*^*tm1*^ transcripts splice from exon 2 to exon 6, skipping the beta-geo insertion. It is documented that a proportion of gene trap alleles produce novel splice products that ‘jump over’ the gene trap. We therefore investigated whether the message detected by WISH *(B)* might result from such a splicing event around the targeted insertion. cDNA was prepared from wild-type, *Pkd1l1*^*+/tm1*^ and *Pkd1l1*^*tm1/tm1*^ 8.5 dpc embryos. PCR primers in exons 1 (5’-TTGGCAGGTGCAACTACTGT-3’) and 6 (5’-CCCATGTTCTTCACTGGGGG-3’) were used to amplify the intervening region. This resulted in a band of the predicted size (~800bp) in wild-type and *Pkd1l1*^*+/tm1*^ samples and a smaller band (~350 bp) in *Pkd1l1*^*+/tm1*^ and *Pkd1l1*^*tm1/tm1*^ samples (gel on right; *t* refers to *tm1*). The wild-type band was missing from the *Pkd1l1*^*tm1/tm1*^ samples. The smaller band is of the size predicted for a splicing event between exons 2 and 6, as confirmed by Sanger sequencing. The resulting message in *Pkd1l1*^*tm1/tm1*^ is out of frame such that any resulting protein would truncate within 18 amino acids of the exon 2-exon 6 splicing event. This would lead to a very small product, lacking all characterised protein domains including having no transmembrane domains. Thus, in *Pkd1l1*^*tm1/tm1*^ embryos, the second exon of *Pkd1l1* either splices onto the beta-geo cassette (which harbours a polyA and stop codon) or splices from exon 2-exon 6 producing an out of frame transcript which contains a stop codon after 18 amino acids. (D) Quantitative analysis of *Pkd1l1* transcripts reveals that the level of transcript detected in 3’ potions of the locus in *Pkd1l1*^*tm1/tm1*^ is equivalent to the level of transcript that splices exons 2–6 in *Pkd1l1*^*tm1/tm1*^ embryos. Only single long *Pkd1l1* Havana-curated transcripts exist in both mouse and humans. To test whether additional start sites might exist we utilised quantitative reverse transcription PCR (qRT-PCR) to measure the expression levels of different regions of the transcript. The following assays were used:
Exon 1–2 assesses expression from the known start site of the locus. Surprisingly, this revealed a 2-fold upregulation of *Pkd1l1* in *Pkd1l1*^*tm1/tm1*^ embryos, suggesting that a negative feedback loop controls *Pkd1l1* expression.Exon 2–3 and Exon 5–6 assess wild-type expression. As exon 3, 4 and 5 are absent from the *Pkd1l1*^*tm1/tm1*^ allele, as expected, no expression in this region of the transcript is evident in *Pkd1l1*^*tm1/tm1*^ mutants.Exon 2-LacZ assesses the splicing from exon 2 into the beta-geo insertion; the predicted splice product. As predicted, this product is present in both *Pkd1l1*^*+/tm1*^ and *Pkd1l1*^*tm1/tm1*^ and is approximately twice as highly expressed in the mutant relative to the heterozygous state.Exon 2–6 assesses splicing around the beta-geo insertion, which is predominant in *Pkd1l1*^*tm1/tm1*^ and *Pkd1l1*^*+/tm1*^ samples. We also observed a very small amount of exon 2–6 splicing in wild-type samples in this assay. However, additional analysis suggests this low level of expression to be an artefact of the qPCR assay and not a biologically meaningful splice variant.Exon 21–22 assesses expression of a 3’ region of the locus. This product overlaps the WISH probe used above *(B)*.
(E) The relative levels of the exon 2–6 versus exon 2-LacZ were calculated, revealing that exon 2–6 splicing occurs at ~35% of the level for exon 2-LacZ splicing in both the *Pkd1l1*^*+/tm1*^ and *Pkd1l1*^*tm1/tm1*^ embryos. In combination with the doubled level of transcription of the *Pkd1l1* locus in *Pkd1l1*^*tm1/tm1*^ embryos, this explains the level of transcript that we detect by exon 21–22 qRT-PCR. For all experiments, error bars show the RQ_min_ and RQ_max_ when confidence levels are set at 95%.
(DOCX)

S2 Fig Pkd1l1^tm1/tm1^ mutants exhibit variable times of death and gross heart and stomach situs defects that are similar to Pkd1l1^rks/rks^ and Dnah11^iv/iv^ mutants. (A-B) Charts showing the observed (Obs) and expected (Exp) frequencies of *Pkd1l1* genotype for embryos dissected at E13.5 *(A)* or recovered as surviving adults *(B)*. There is a statistically significant loss of *Pkd1l1*^*tm1/tm1*^ mutants at these time points (chi-square test applied). When dissected at E13.5, 32% of *Pkd1l1*^*tm1/tm1*^ (n = 13/41) had already arrested *in utero* (at various times between E9.5-E12.5). Approximately 35% of the expected number of homozygotes survived until adulthood. (C-D) Examples of reversed heart (H) and stomach (S) laterality in *Pkd1l1*^*tm1/tm1*^ embryos *(D)* compared to a control *(C)* at E13.5. Normally, the heart apex and stomach are positioned to the left of the body cavity, but this is reversed in a proportion of *Pkd1l1*^*tm1/tm1*^ mutants. R-L refers to right-left. (E) Heart and stomach laterality scored at E13.5 for *Pkd1l1*^*tm1/tm1*^, *Dnah11*^*iv/iv*^ and *Pkd1l1*^*rks/rks*^ mutants. The percentage of embryos showing each phenotype and the total number of embryos examined is given. *t* refers to *Pkd1l1*^*tm1*^.
(DOCX)

S3 Fig Quantitation of nodal cilia rotation frequency in *Pkd1l1*^*tm1/tm1*^ and control embryos. Cilia rotation fequency for *Pkd1l1*^*tm1/tm1*^ mutants and wild-type controls. At least three embryos of each genotype were assessed and analysis was performed blind to genotype. Error bars represent standard error of the mean. No statistically significant difference (ns) was found between the two genotypes, Student *t*-test applied.
(DOCX)

S4 Fig PIV analysis of nodal flow. PIV analysis was conducted on of wild-type (WT), *Pkd1l1*^*tm1/tm1*^, *Pkd2*^*lrm4/lrm4*^, *Pkd1l1*^*rks/rks*^ and *Dnah11*^*iv/iv*^ 8.5 dpc embryos. Examples of PIV analysis at different somite stages (ss) are shown. Flow was present and leftward at all stages assessed in all genotypes except *Dnah11*^*iv/iv*^ which exhibited absence of flow.
(DOCX)

S5 Fig Overall *Cerl2* levels are decreased in *Pkd1l1*^*tm1/tm1*^ mutants compared to control embryos. *Cerl2* expression at the node of *Pkd1l1*^*tm1/tm1*^ mutants and control embryos at the 1–3 somite stage from three separate litters. In each case, expression is more symmetrical and expression levels are lower in *Pkd1l1*^*tm1/tm1*^ embryos than in controls. Embryos from the same litter were treated identically throughout the procedure and scoring of expression was performed prior to genotyping.
(DOCX)

S1 Table Genetic interaction between *Dnah11*^*iv*^ and *Pkd1l1*^*rks*^ or *Pkd2*^*lrm4*^. n, number; NS, normal situs; RS, reversed situs; RI, right isomerism; LI, left isomerism; PI, partial isomerism; L, left; R, right; B, bilateral; A, absent. Lung situs was scored at 13.5 dpc, while LPM *Pitx2* expression was determined by WISH at 8.5 dpc.
(DOCX)

S2 Table Genetic interaction between *Pkd1l1*^*tm1*^ and *Pkd2*^*lrm4*^. n, number; NS, normal situs; RS, reversed situs; RI, right isomerism; LI, left isomerism; PI, partial isomerism. Lung situs was scored at 13.5 dpc.
(DOCX)

S3 Table Genetic interaction between *Kif3a*^*–*^ and *Pkd1l1*^*rks*^ or *Pkd2*^*lrm4*^. n, number; L, left; R, right; B, bilateral; A, absent. LPM *Pitx2* expression was determined by WISH at 8.5 dpc.
(DOCX)

S4 Table *Pkd1l1*^*tm1*^ primers used for qRT-PCR. (DOCX)

S5 Table Numerical results of flow chamber experiments. (DOCX)
