# Supplementary figures and images for: Genetic Analysis Reveals a Hierarchy of Interactions between Polycystin-Encoding Genes and Genes Controlling Cilia Function during Left-Right Determination
Source: PLoS Genet. 2016 Jun 6;12(6):e1006070. doi: 10.1371/journal.pgen.1006070 (PMC4894641; doi:10.1371/journal.pgen.1006070)

# S1 Fig.

**Characterisation of the *Pkd1l1tm1* allele.**


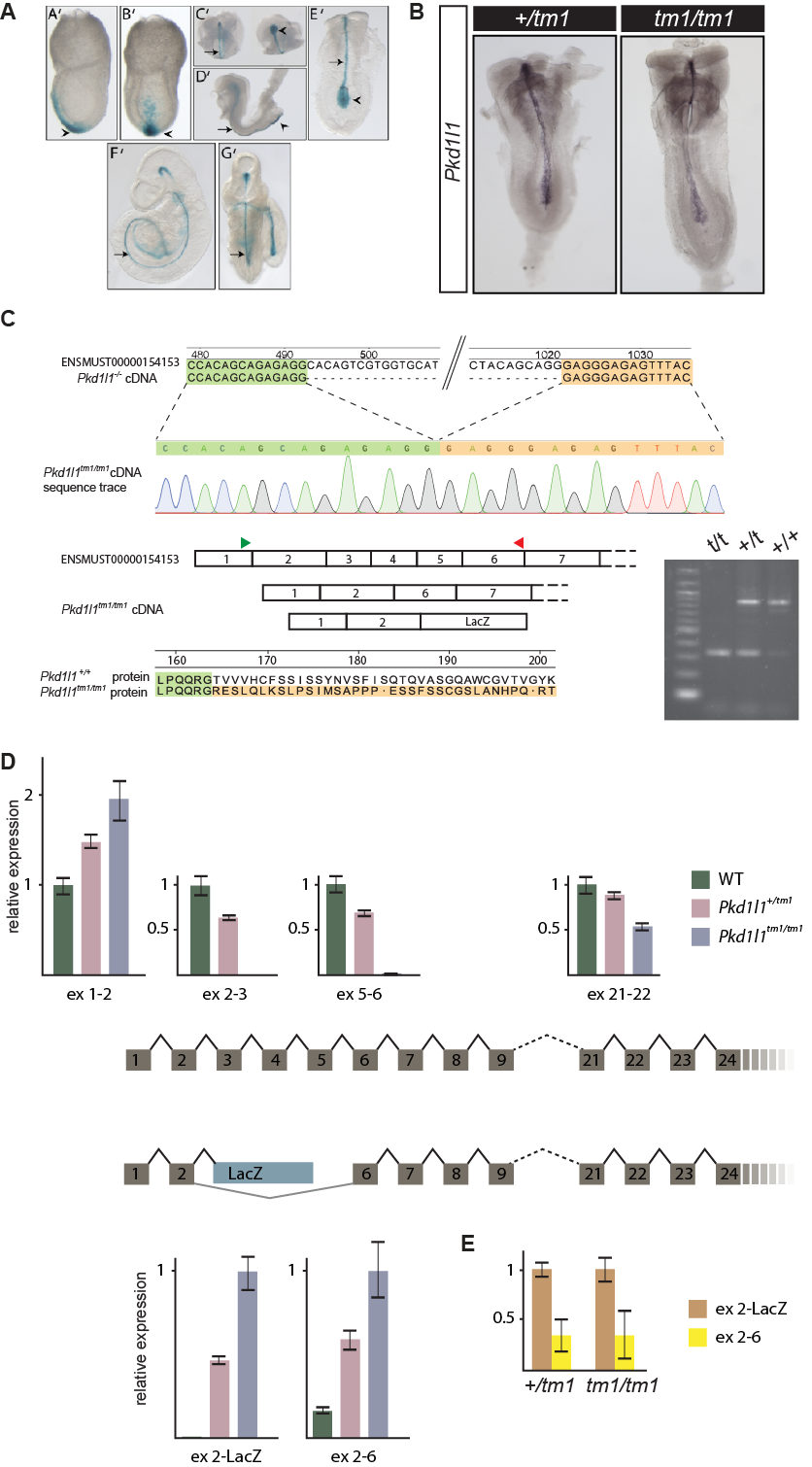

Supplement: S1 Fig — The Pkd1l1tm1 allele (Pkd1l1tm1Lex; [33]) comprises a beta-galactosidase-neomycin fusion gene (beta-geo) inserted in place of exons 3, 4 and 5 (labelled ‘LacZ’ in the schematic given in D). Importantly, this insertion contains a stop codon and polyA signal at its 3’ end. The data in this figure demonstrate that in Pkd1l1tm1/tm1 mutants, the gene is disrupted either by splicing onto the beta-geo cassette or by splicing around the cassette in a fashion that introduces a premature stop codon which truncates the protein very early, suggesting that Pkd1l1tm1 is a null or strong hypomorph. (AA’-AG’) Expression of Pkd1l1 was assessed by LacZ staining, revealing that expression from the beta-geo reporter locus mirrors the endogenous expression pattern [16]. Pkd1l1+/tm1 embryos that were phenotypically normal; 7.5 dpc (AA’-AB’), 8.5 dpc (AC’-AE’), and 9.5 dpc (AF’-AG’) embryos were assessed. (B) WISH analysis of Pkd1l1 expression in Pkd1l1+/tm1 and Pkd1l1tm1/tm1 embryos at 8.5 dpc. If all transcripts splice into beta-geo, then we would predict there to be no mRNA present for 3’ portions of Pkd1l1. However, WISH revealed equivalent expression patterns in both wild-type and mutant embryos (assessed with a probe covering exons 20–24); expression in the Pkd1l1tm1/tm1 embryos appears slightly reduced. (C) A proportion of Pkd1l1tm1 transcripts splice from exon 2 to exon 6, skipping the beta-geo insertion. It is documented that a proportion of gene trap alleles produce novel splice products that ‘jump over’ the gene trap. We therefore investigated whether the message detected by WISH (B) might result from such a splicing event around the targeted insertion. cDNA was prepared from wild-type, Pkd1l1+/tm1 and Pkd1l1tm1/tm1 8.5 dpc embryos. PCR primers in exons 1 (5’-TTGGCAGGTGCAACTACTGT-3’) and 6 (5’-CCCATGTTCTTCACTGGGGG-3’) were used to amplify the intervening region. This resulted in a band of the predicted size (~800bp) in wild-type and Pkd1l1+/tm1 samples and a smaller band (~350 [file pgen.1006070.s001.docx]

## S3 Fig.

**Quantitation of nodal cilia rotation frequency in *Pkd1l1tm1/tm1* and control embryos.**


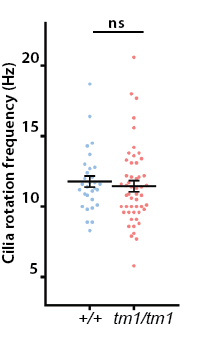

Supplement: S3 Fig — Cilia rotation fequency for Pkd1l1tm1/tm1 mutants and wild-type controls. At least three embryos of each genotype were assessed and analysis was performed blind to genotype. Error bars represent standard error of the mean. No statistically significant difference (ns) was found between the two genotypes, Student t-test applied. (DOCX) [file pgen.1006070.s003.docx]

# S4 Fig.

**PIV analysis of nodal flow**


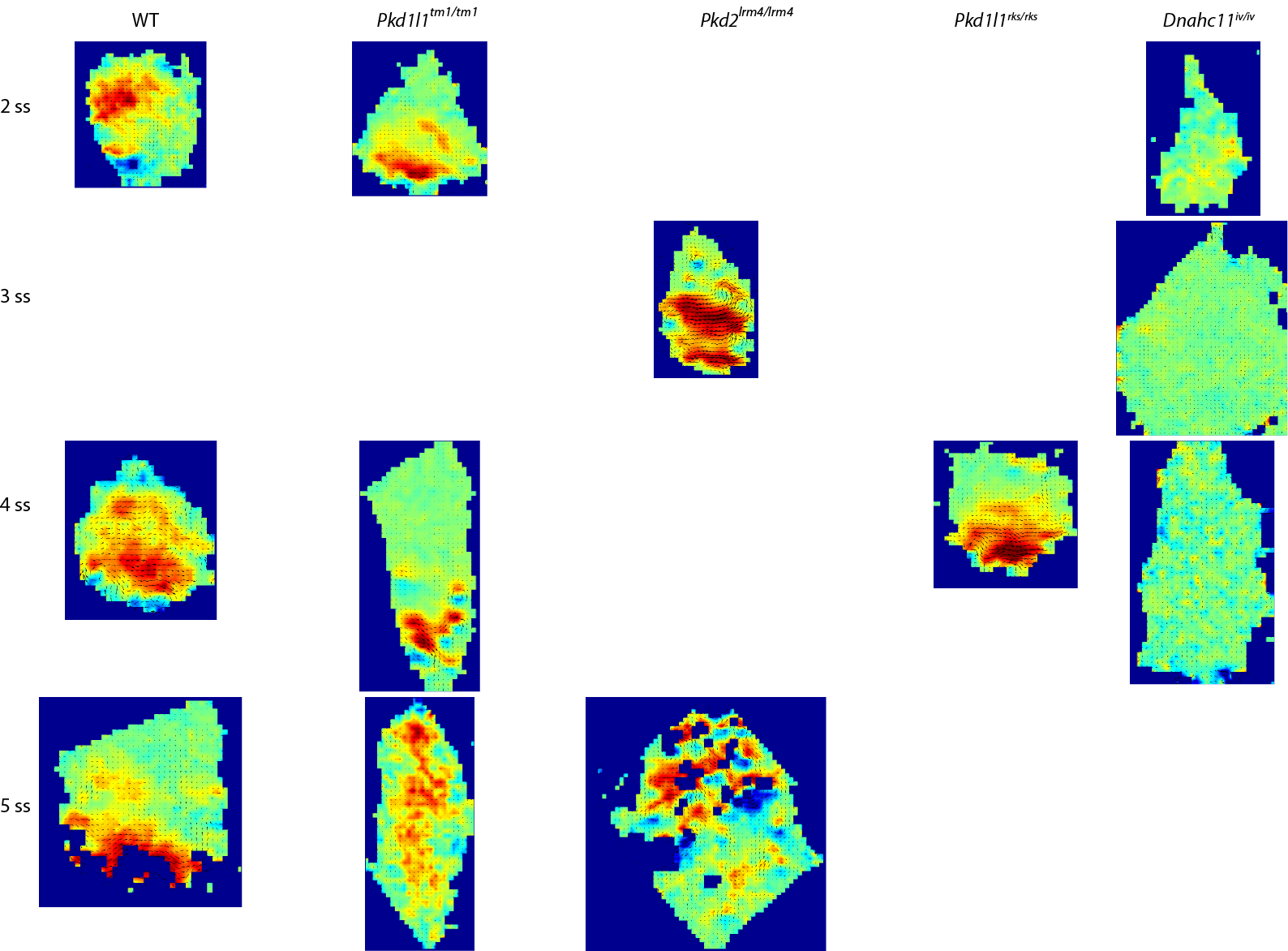

Supplement: S4 Fig — PIV analysis was conducted on of wild-type (WT), Pkd1l1tm1/tm1, Pkd2lrm4/lrm4, Pkd1l1rks/rks and Dnah11iv/iv 8.5 dpc embryos. Examples of PIV analysis at different somite stages (ss) are shown. Flow was present and leftward at all stages assessed in all genotypes except Dnah11iv/iv which exhibited absence of flow. (DOCX) [file pgen.1006070.s004.docx]

## S5 Fig.

**Overall *Cerl2* levels are decreased in *Pkd1l1tm1/tm1* mutants compared to control embryos.**


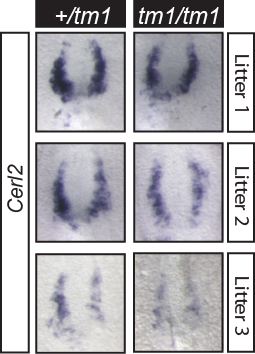

Supplement: S5 Fig — Cerl2 expression at the node of Pkd1l1tm1/tm1 mutants and control embryos at the 1–3 somite stage from three separate litters. In each case, expression is more symmetrical and expression levels are lower in Pkd1l1tm1/tm1 embryos than in controls. Embryos from the same litter were treated identically throughout the procedure and scoring of expression was performed prior to genotyping. (DOCX) [file pgen.1006070.s005.docx]
