# Supplementary material for: Genetic Analysis Reveals a Hierarchy of Interactions between Polycystin-Encoding Genes and Genes Controlling Cilia Function during Left-Right Determination
Source: PLoS Genet. 2016 Jun 6;12(6):e1006070. doi: 10.1371/journal.pgen.1006070 (PMC4894641; doi:10.1371/journal.pgen.1006070)

# S2 Fig.

***Pkd1l1^tm1/tm1^* mutants exhibit variable times of death and gross heart and stomach situs defects that are similar to *Pkd1l1^rks/rks^* and *Dnah11^iv/iv^* mutants.**


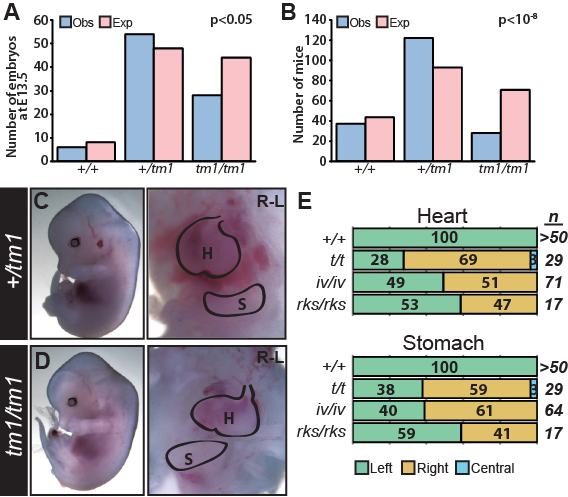

Supplement: S2 Fig — (A-B) Charts showing the observed (Obs) and expected (Exp) frequencies of Pkd1l1 genotype for embryos dissected at E13.5 (A) or recovered as surviving adults (B). There is a statistically significant loss of Pkd1l1tm1/tm1 mutants at these time points (chi-square test applied). When dissected at E13.5, 32% of Pkd1l1tm1/tm1 (n = 13/41) had already arrested in utero (at various times between E9.5-E12.5). Approximately 35% of the expected number of homozygotes survived until adulthood. (C-D) Examples of reversed heart (H) and stomach (S) laterality in Pkd1l1tm1/tm1 embryos (D) compared to a control (C) at E13.5. Normally, the heart apex and stomach are positioned to the left of the body cavity, but this is reversed in a proportion of Pkd1l1tm1/tm1 mutants. R-L refers to right-left. (E) Heart and stomach laterality scored at E13.5 for Pkd1l1tm1/tm1, Dnah11iv/iv and Pkd1l1rks/rks mutants. The percentage of embryos showing each phenotype and the total number of embryos examined is given. t refers to Pkd1l1tm1. (DOCX) [file pgen.1006070.s002.docx]
