# Supplementary material for: Genetic Analysis Reveals a Hierarchy of Interactions between Polycystin-Encoding Genes and Genes Controlling Cilia Function during Left-Right Determination
Source: PLoS Genet. 2016 Jun 6;12(6):e1006070. doi: 10.1371/journal.pgen.1006070 (PMC4894641; doi:10.1371/journal.pgen.1006070)
Supplement: S1 Table — n, number; NS, normal situs; RS, reversed situs; RI, right isomerism; LI, left isomerism; PI, partial isomerism; L, left; R, right; B, bilateral; A, absent. Lung situs was scored at 13.5 dpc, while LPM Pitx2 expression was determined by WISH at 8.5 dpc. (DOCX) [file pgen.1006070.s006.docx]

**S1 Table: Genetic interaction between *Dnahc11^iv^* and *Pkd1l1^rks^* or *Pkd2^lrm4^***

| Genotype | |  | Lungs | | | | | |  | *Pitx2* | | | | |
| --- | --- | --- | --- | --- | --- | --- | --- | --- | --- | --- | --- | --- | --- | --- |
| *Dnahc11* | *Pkd1l1/Pkd2* |  | n | NS | RS | RI | LI | PI |  | n | L | R | B | A |
| *+/+* | *+/+* |  | 5 | 100 | 0.0 | 0.0 | 0.0 | 0.0 |  | 5 | 100 | 0.0 | 0.0 | 0.0 |
| *+/iv* | *+/rks* |  | 18 | 100 | 0.0 | 0.0 | 0.0 | 0.0 |  | 7 | 100 | 0.0 | 0.0 | 0.0 |
| *+/iv* | *rks/rks* |  | 8 | 12.5 | 0.0 | 87.5 | 0.0 | 0.0 |  | 2 | 0.0 | 0.0 | 0.0 | 100 |
| *iv/iv* | *+/rks* |  | 14 | 28.6 | 42.9 | 0.0 | 28.6 | 0.0 |  | 7 | 42.9 | 0.0 | 28.6 | 28.6 |
| *iv/iv* | *rks/rks* |  | 9 | 0.0 | 0.0 | 100 | 0.0 | 0.0 |  | 4 | 0.0 | 0.0 | 0.0 | 100.0 |
| *+/iv* | *+/lrm4* |  | 16 | 100 | 0.0 | 0.0 | 0.0 | 0.0 |  | 17 | 100 | 0.0 | 0.0 | 0.0 |
| *+/iv* | *lrm4/lrm4* |  | 6 | 0.0 | 0.0 | 100 | 0.0 | 0.0 |  | 8 | 12.5 | 0.0 | 0.0 | 87.5 |
| *iv/iv* | *+/lrm4* |  | 9 | 44.4 | 44.4 | 0.0 | 11.1 | 0.0 |  | 15 | 13.3 | 20.0 | 53.3 | 13.3 |
| *iv/iv* | *lrm4/lrm4* |  | 5 | 0.0 | 0.0 | 100 | 0.0 | 0.0 |  | 7 | 0.0 | 0.0 | 0.0 | 100 |

n, number; NS, normal situs; RS, reversed situs; RI, right isomerism; LI, left isomerism; PI, partial isomerism; L, left; R, right; B, bilateral; A, absent. Lung situs was scored at 13.5 dpc, while LPM *Pitx2* expression was determined by WISH at 8.5 dpc.
