# Supplementary material for: Genetic Analysis Reveals a Hierarchy of Interactions between Polycystin-Encoding Genes and Genes Controlling Cilia Function during Left-Right Determination
Source: PLoS Genet. 2016 Jun 6;12(6):e1006070. doi: 10.1371/journal.pgen.1006070 (PMC4894641; doi:10.1371/journal.pgen.1006070)
Supplement: S2 Table — n, number; NS, normal situs; RS, reversed situs; RI, right isomerism; LI, left isomerism; PI, partial isomerism. Lung situs was scored at 13.5 dpc. (DOCX) [file pgen.1006070.s007.docx]

**S2 Table: Genetic interaction between *Pkd1l1^tm1^* and *Pkd2^lrm4^***

| Genotype | |  | Lungs | | | | | |
| --- | --- | --- | --- | --- | --- | --- | --- | --- |
| *Pkd2* | *Pkd1l1* |  | n | NS | RS | RI | LI | PI |
| *+/+* | *+/+* |  | 1 | 100 | 0.0 | 0.0 | 0.0 | 0.0 |
| *+/+* | *+/tm1* |  | 4 | 100 | 0.0 | 0.0 | 0.0 | 0.0 |
| *+/+* | *tm1/tm1* |  | 2 | 100 | 0.0 | 0.0 | 0.0 | 0.0 |
| *+/lrm4* | *+/+* |  | 9 | 100 | 0.0 | 0.0 | 0.0 | 0.0 |
| *+/lrm4* | *+/ m1* |  | 13 | 100 | 0.0 | 0.0 | 0.0 | 0.0 |
| *+/lrm4* | *tm1/tm1* |  | 2 | 0.0 | 0.0 | 0.0 | 100 | 0.0 |
| *lrm4/lrm4* | *+/+* |  | 3 | 0.0 | 0.0 | 100 | 0.0 | 0.0 |
| *lrm4/lrm4* | *+/tm1* |  | 2 | 0.0 | 0.0 | 100 | 0.0 | 0.0 |
| *lrm4/lrm4* | *tm1/tm1* |  | 4 | 0.0 | 0.0 | 100 | 0.0 | 0.0 |

n, number; NS, normal situs; RS, reversed situs; RI, right isomerism; LI, left isomerism; PI, partial isomerism. Lung situs was scored at 13.5 dpc.
