# Supplementary material for: Genetic Analysis Reveals a Hierarchy of Interactions between Polycystin-Encoding Genes and Genes Controlling Cilia Function during Left-Right Determination
Source: PLoS Genet. 2016 Jun 6;12(6):e1006070. doi: 10.1371/journal.pgen.1006070 (PMC4894641; doi:10.1371/journal.pgen.1006070)
Supplement: S3 Table — n, number; L, left; R, right; B, bilateral; A, absent. LPM Pitx2 expression was determined by WISH at 8.5 dpc. (DOCX) [file pgen.1006070.s008.docx]

**S3 Table: Genetic interaction between *Kif3a^–^* and *Pkd1l1^rks^* or *Pkd2^lrm4^***

| Genotype | |  | *Pitx2* | | | | |
| --- | --- | --- | --- | --- | --- | --- | --- |
| *Kif3a* | *Pkd1l1/Pkd2* |  | n | L | R | B | A |
| *+/+* | *+/+* |  | 7 | 100 | 0.0 | 0.0 | 0.0 |
| *+/-* | *+/+* |  | 21 | 100 | 0.0 | 0.0 | 0.0 |
| *–/–* | *+/+* |  | 18 | 5.5 | 0.0 | 94.4 | 0.0 |
| *+/+* | *+/rks* |  | 6 | 100 | 0.0 | 0.0 | 0.0 |
| *+/-* | *+/rks* |  | 30 | 100 | 0.0 | 0.0 | 0.0 |
| *–/–* | *+/rks* |  | 5 | 20.0 | 0.0 | 80.0 | 0.0 |
| *+/+* | *rks/rks* |  | 6 | 0.0 | 0.0 | 0.0 | 100 |
| *+/-* | *rks/rks* |  | 7 | 42.9 | 14.3 | 0.0 | 57.1 |
| *–/–* | *rks/rks* |  | 2 | 0.0 | 0.0 | 100 | 0.0 |
| *+/+* | *+/lrm4* |  | 14 | 100 | 0.0 | 0.0 | 0.0 |
| *+/-* | *+/lrm4* |  | 27 | 96.3 | 0.0 | 0.0 | 3.7 |
| *–/–* | *+/lrm4* |  | 17 | 0.0 | 0.0 | 100 | 0.0 |
| *+/+* | *lrm4/lrm4* |  | 6 | 0.0 | 0.0 | 0.0 | 100 |
| *+/-* | *lrm4/lrm4* |  | 9 | 0.0 | 0.0 | 0.0 | 100 |
| *–/–* | *lrm4/lrm4* |  | 5 | 0.0 | 0.0 | 100 | 0.0 |

n, number; L, left; R, right; B, bilateral; A, absent. LPM *Pitx2* expression was determined by WISH at 8.5 dpc.
