# Supplementary material for: Genetic Analysis Reveals a Hierarchy of Interactions between Polycystin-Encoding Genes and Genes Controlling Cilia Function during Left-Right Determination
Source: PLoS Genet. 2016 Jun 6;12(6):e1006070. doi: 10.1371/journal.pgen.1006070 (PMC4894641; doi:10.1371/journal.pgen.1006070)
Supplement: S4 Table — (DOCX) [file pgen.1006070.s009.docx]

**S4 Table: *Pkd1l1^tm1Lex^* primers used for qRT-PCR**

| **Amplified exons** | **Forward Primer Sequence** | **Reverse Primer Sequence** |
| --- | --- | --- |
| 1-2 | GCAACTACTGTGACTGGCTACCA | TCGGCTCTATGAAAGTCATGGA |
| 2-3 | CCATGATAGCGAAGCTCTTTCC | GAAATAAAGGACACATTGTATGAAGAAATG |
| 5-6 | CAGCTCCTCCAGCCATGTCT | GGGTAGTGGAGCTGACATTATTGA |
| 21-22 | TCAGCCAGCTCATGTTCTTCA | CATAGTCACAGGTGTCTCCAGTTGT |
| 2-lacZ | CCATGATAGCGAAGCTCTTTCC | GGCCGCTATGGCCTCTAGA |
| 2-6 | CCATGATAGCGAAGCTCTTTCC | GGGTAGTGGAGCTGACATTATTGA |
