# Supplementary material for: Genetic Analysis Reveals a Hierarchy of Interactions between Polycystin-Encoding Genes and Genes Controlling Cilia Function during Left-Right Determination
Source: PLoS Genet. 2016 Jun 6;12(6):e1006070. doi: 10.1371/journal.pgen.1006070 (PMC4894641; doi:10.1371/journal.pgen.1006070)
Supplement: S5 Table — (DOCX) [file pgen.1006070.s010.docx]

**S5 Table: Numerical results of flow chamber experiments**

| **Supplemental Table on Flow-induced calcium signaling** | | | |
| --- | --- | --- | --- |
| **Cell type** | **^1^Transfection** | **^2^GFP (-)** | **^3^GFP (+)** |
| **Wild-type** | **control** | 151.94±8.04 | 163.51±12.20 |
|  | ***Pkd1l1*** | 161.50±1.96 | 185.45±6.13 |
|  | ***Pkd1l1^rks^*** | 156.61±11.51 | 161.98±7.97 |
| **Pkd1^-/-^** | **control** | 99.97±4.06 | 99.90±4.06 |
|  | ***Pkd1l1*** | 103.29±4.66 | 127.33±7.40 |
|  | ***Pkd1l1^rks^*** | 100.71±2.07 | 100.92±0.54 |

Note:

- All values are expressed in mean ± standard error of mean

- Values of calcium signaling in response to fluid-shear stress are in arbitrary units normalized to the baseline value prior to fluid flow

- **^1^**control = empty vector (negative control)

- **^2^**GFP (-) = non-transfected cells (internal control)

- **^3^**GFP (+) = transfected cells
